# Stage-Dependent Effects of “Too Little and Too Much” Medial Prefrontal Activity on Reversal Learning in Rats: Functional Inhibition Impairs Early, Whereas Neural Disinhibition Impairs Late Reversals

**DOI:** 10.1523/JNEUROSCI.1930-25.2026

**Published:** 2026-08-13

**Authors:** Jacco G. Renström, Charlotte J. L. Taylor, Rachel Grasmeder Allen, Luke O’Hara, Joanna Loayza, Jacob Juty, Paula M. Moran, Moritz von Heimendahl, Serena Deiana, Johann Du Hoffman, Carl W. Stevenson, Silvia Maggi, Tobias Bast

**Affiliations:** ^1^School of Psychology, University of Nottingham, Nottingham NG7 2RD, United Kingdom; ^2^Boehringer Ingelheim Pharma GmbH & Co. KG, Biberach 88400, Germany; ^3^School of Biosciences, University of Nottingham, Nottingham LE12 5RD, United Kingdom

**Keywords:** DREADD, GABAergic inhibition, medial prefrontal cortex (mPFC), neural disinhibition, reversal learning, schizophrenia

## Abstract

Schizophrenia is associated with prefrontal cortex dysfunction, including neural disinhibition (reduced GABAergic inhibition) and hypoactivation (“hypofrontality”), alongside impaired reversal learning. However, evidence implicating prefrontal regions, including the rodent medial PFC (mPFC), in reversal learning is mixed. mPFC involvement may scale with demand for mPFC-dependent attention and cognitive control to overcome prepotent responses and to learn that reward contingencies can reverse, which is highest during early reversals. We therefore hypothesized that (1) the mPFC is required for early reversal learning but less important when reversal proficiency is high during late reversals. Furthermore, mPFC disinhibition may impair cognitive performance by causing circuit-level disruption outside the mPFC. Therefore, we hypothesized that (2) even when the mPFC is not required, mPFC disinhibition may impair reversal performance. To test Hypotheses 1 and 2, we first examined the effect of mPFC functional inhibition and disinhibition, by microinfusion of the GABA-A receptor agonist muscimol and antagonist picrotoxin, on early reversals (reversals 1–3) versus late reversals (reversal 5 onward) on a 2-lever discrimination task in adult male rats. mPFC functional inhibition by muscimol impaired only early reversals, increasing perseveration and impairing lose-shift behavior at reversal 2. In contrast, mPFC disinhibition by picrotoxin impaired late reversals, reducing lose-shift and win-stay behavior. Using chemogenetic mPFC disinhibition (hM4Di-mediated inhibition of GABAergic neurons), we further tested Hypothesis 2. Similar to mPFC picrotoxin, chemogenetic mPFC disinhibition impaired late reversals, primarily disrupting win-stay behavior. Our findings suggest that reduced and disinhibited mPFC activity impair distinct aspects of reversal learning.

## Significance Statement

Schizophrenia is associated with reduced activation (“hypofrontality”) and neural disinhibition (reduced GABAergic inhibition) within the prefrontal cortex (PFC). However, it is not clear if and how these distinct aspects of prefrontal dysfunction contribute to impaired reversal learning, a key feature of the cognitive inflexibility characterizing schizophrenia. Here, we combined bidirectional manipulations of prefrontal GABAergic inhibition with testing of reversal learning in rats. Increasing prefrontal functional inhibition (i.e., reducing prefrontal activation) selectively impaired early reversals, enhancing perseveration and reducing lose-shift behavior, whereas prefrontal disinhibition disrupted late reversals, impairing both lose-shift and win-stay behavior. Our findings suggest that reduced activation and disinhibition of PFC disrupt distinct aspects of reversal learning, by distinct mechanisms.

## Introduction

Schizophrenia is associated with imbalanced neural activity in the dorsolateral prefrontal cortex (dlPFC), specifically, reduced activation (hypofrontality; Carter et al., 1998; Minzenberg et al., 2009) and reduced GABAergic inhibition (neural disinhibition; Schoonover et al., 2020). These abnormalities were suggested to be causally linked, whereby hyperexcitability due to neural disinhibition at early disease stages contributes to hypofrontality at later stages (Krystal and Anticevic, 2015). Research in animal models showed that inhibited and disinhibited prefrontal activity, characterized by reduced and enhanced burst firing, respectively (Pezze et al., 2014), caused cognitive impairments relevant to schizophrenia and other neuropsychiatric disorders (Tse et al., 2015; Bast et al., 2017).

Reversal learning deficits are one marked and robust cognitive impairment in schizophrenia (Murray et al., 2008; Leeson et al., 2009). Reversal learning involves the reversal of response–reward associations, whereby a previously rewarded response is no longer rewarded whereas a previously unrewarded response becomes rewarded. This process has mainly been associated with the orbitofrontal cortex (OFC), with less importance ascribed to the primate dlPFC or rodent medial PFC (mPFC; Chudasama and Robbins, 2003; McAlonan and Brown, 2003; Izquierdo et al., 2004, 2017; Boulougouris et al., 2007; Murray et al., 2007; Floresco et al., 2008; Hervig et al., 2020), two regions supporting executive functions across species (Brown and Bowman, 2002; Uylings et al., 2003; Dalley et al., 2004; Seamans et al., 2008; Laubach et al., 2018; Izquierdo, 2024).

Nevertheless, imbalanced mPFC activity may impair reversal learning, whereby reduced and disinhibited mPFC activity may cause distinct impairments at different reversal stages. During early reversals, subjects have yet to learn that reversals occur and have a strong bias for the established response; in contrast, during late reversals, subjects reverse their response proficiently (Jang et al., 2015; Izquierdo et al., 2017). In previous studies, mPFC lesions impaired reversal learning on attentionally demanding paradigms, requiring difficult discriminations or simultaneous monitoring of multiple discriminanda (Bussey et al., 1997; Chudasama and Robbins, 2003; Brigman and Rothblat, 2008; Kosaki and Watanabe, 2012). This is most relevant to early reversals, when subjects first ascribe relevance to task-pertinent stimuli and form attentional sets. Moreover, the mPFC is required to overcome prepotent behavioral responses (Miller and Cohen, 2001; Haddon and Killcross, 2007; Marquis et al., 2007), which may be most important during early reversal stages, before subjects learn about the possibility of repeated reversals. In contrast, the mPFC may be less required during late reversals, when demands for mPFC-dependent attention and response suppression are reduced. However, even when the mPFC is not required, mPFC disinhibition may still impair reversal learning by aberrant mPFC activity disrupting processing in mPFC projection sites (Auger and Floresco, 2014; Bast et al., 2017; Auger et al., 2019), which include the OFC (Sesack et al., 1989). Therefore, we hypothesized that (1) reduced mPFC activity will mainly impair early reversals but affect late reversals less; (2) even when the mPFC is not required (e.g., during late reversals), mPFC disinhibition may impair reversals by disrupting activity in task-relevant mPFC projection sites (e.g., OFC; although we did not examine relevant projection sites).

To test Hypotheses 1 and 2, we examined how bidirectional changes in mPFC activity, through functional inhibition or disinhibition by microinfusion of the GABA-A receptor agonist muscimol or antagonist picrotoxin, respectively (Pezze et al., 2014), affect early reversals (reversal 1–3) and late reversals (reversal 5 onward) in rats, using a food-reinforced two-lever discrimination task (Brady and Floresco, 2015; Gonçalves et al., 2023; Reversal experiment 1 and 2). We found that mPFC inhibition impaired early, whereas disinhibition impaired late, reversals. To further address Hypothesis 2, we examined the impact of chemogenetic mPFC disinhibition on late reversals (Reversal experiment 3). The chemogenetic mPFC disinhibition used Vgat (vesicular GABA transporter)-dependent expression of the inhibitory hM4Di DREADD (Designer Receptor Exclusively Activated by Designer Drugs; Roth, 2016) in Vgat-Cre rats to inhibit mPFC GABAergic neurons. We also report histological, in vivo electrophysiological and locomotor validation of this novel chemogenetic model.

## Materials and Methods

### Rats

This study included only male rats, because the stereotaxic coordinates and infusion parameters used were established in male rats (Pezze et al., 2014). Male and female rats have shown similar reversal learning abilities, although some of their behaviors during reversal learning session may subtly differ (Aguirre et al., 2026). Although we expect the neurobehavioral processes examined in the present study to be qualitatively similar across sexes, any sex differences could be examined in future studies. In total, we used 101 young adult male rats, all supplied by Envigo. This included two cohorts of Lister hooded rats for the mPFC microinfusion studies; one cohort of Lister hooded rats for a control study on the effect of the DREADD ligand clozapine-*N*-oxide (CNO) on reversal learning; and two cohorts of Vgat-Cre rats for the chemogenetic studies (Table 1). The Vgat-Cre rats [homozygous LE-VGATem1(IRES-Cre)Sage; https://www.inotiv.com/research-model/hsdsage-le-vgatem1-ires-cre-sage] are Long–Evans hooded rats with a knock-in of Cre-recombinase under control of the endogenous VGAT (vesicular GABA transporter) promoter, to enable Cre expression in GABAergic neurons. Lister hooded rats weighed between 290 and 340 g (8–9 weeks), and Vgat-Cre rats weighed 300–460 g (8–11 weeks old) at surgery (cannula implantation or viral injection, respectively). Seven out of 64 Lister hooded rats used for the microinfusion studies had to be excluded for various reasons (Table 1, Cohorts #1 and 2). Two of the overall 21 Vgat-Cre rats (Table 1, Cohorts #4 and #5) had to be excluded due to convulsive seizures, which were observed after viral vector injection and before any CNO injections. One additional Vgat-Cre rat in Cohort #4 showed less pronounced seizures, which did not require killing or exclusion from testing and analysis. Two additional Vgat-Cre rats were excluded from the electrophysiological characterization involving the injection of 6.0 mg/kg CNO-2HCl, because the electrodes were located too anterior and there was insufficient DREADD expression around the electrode tips; brain sections of these two rats, which showed strong mPFC DREADD expression, could still be included in the histological analyses. For sample size justifications, see sections below outlining the different experiments.

**Table 1.** Rat cohorts used in the present study

Cohort	Initial *n*, strain	Objectives	Rats excluded	Experiments performed, design, final *n*
1	*n* = 48 (2× *n* = 24), Lister hooded	Impact of mPFC picrotoxin, muscimol, or saline on early reversals	One died during surgery; one killed due to cannula complications; two incomplete testing	Reversal experiment 1: early reversal learning, between-subjects, *n* = 44; saline, *n* = 15; picrotoxin, *n* = 14; muscimol, *n* = 15
2	*n* = 16, Lister hooded	Impact of mPFC picrotoxin, muscimol, or saline on late reversals	Three killed due to cannula complications	Reversal experiment 2: late reversal learning, within-subjects, *n* = 13
3	*n* = 8, VGAT-Cre	mPFC chemogenetic disinhibition—histological and in vivo electrophysiological characterization	One killed due to repeated seizures; two removed from electrophysiological comparison due to low DREADD expression	Chemogenetics validation: in vivo electrophysiology, 6.0 mg/kg CNO-2HCl versus saline, within-subjects, *n* = 5
				2. Histological characterization (DREADD penetrance and selectivity), *n* = 6
4	*n* = 13, VGAT-Cre	mPFC chemogenetic disinhibition: impact on late reversals and locomotor activity, and in vivo electrophysiological characterization	One killed due to repeated seizures	Reversal experiment 3: late reversal learning, 6.0, 1.5, 3.0, 4.5 mg/kg CNO-2HCl versus saline, within-subjects, *n* = 12
				2. Chemogenetics validation: locomotor activity, 4.5 mg/kg CNO-2HCl versus saline, within-subjects, *n* = 12
				3. Chemogenetic validation: in vivo electrophysiology, 4.5 mg/kg CNO-2HCl versus saline, within-subjects, *n* = 5
5	*n* = 16, Lister hooded	Control for CNO effects on late reversals in WT rats without DREADD	No rats excluded	Pretrained on late reversals to R10 as part of separate behavioral study (not reported in manuscript)
				2. Reversal experiment 3 (control): late reversal learning, 4.5 mg/kg CNO-2HCl versus saline, within-subjects, *n* = 16

Five cohorts of rats were used to address different objectives. Initial rat numbers (*n*), exclusions, and resulting final *n* after exclusions are shown. Experiments in Cohort #1 were run in two batches of *n* = 24 rats (total *n* = 48). Note: one additional Vgat-Cre rat (Cohort #4) showed seizures, which did not require killing and, therefore, was not excluded.

Rats were housed in groups of three or four, in individually ventilated “Double Decker” cages (462 mm × 403 mm × 404 mm; Tecniplast) with temperature and humidity control (21 ± 1.5°C, 50 ± 8%) and a 12 h light/dark cycle (lights on at 0700). All experimental procedures were carried out during the light phase. Rats had *ad libitum* access to water throughout the study. Access to food (Teklad Global 18% Protein Rodent Diet 2018C; Envigo) was *ad libitum* until the start of food restriction 1 d prior to the start of operant pretraining. During food restriction, rats received daily food rations of 15–21 g per rat and were weighed daily to maintain body weights at 85–90% of free-feeding weights. On pretraining and test days, rats were weighed before the day's operant task session and received their daily food ration after completing the session (in addition to the food reward received during the session). All in vivo procedures were conducted in the Biological Support Unit (BSU) at the University of Nottingham in accordance with the United Kingdom (UK) Animals (Scientific Procedures) Act 1986, approved by the University of Nottingham's Animal Welfare and Ethical Review Body and run under the authority of Home Office project license PP1257468. Methods and results are reported in line with the ARRIVE guidelines (Lilley et al., 2020).

### mPFC functional inhibition and disinhibition by drug microinfusions

#### Guide cannula implantation into mPFC for microinfusions

Guide cannulae were implanted into the mPFC using the same coordinates and similar methods as described in detail in our previous studies (Pezze et al., 2014). Two small holes were drilled through the skull at the following coordinates: +3 mm anterior and ±0.6 mm lateral from bregma. Bilateral infusion guide cannulae (“mouse” model C235GS-5-1.2; Plastics One, Bilaney Consultants) were lowered to 3.5 mm ventral from the skull surface and secured to the skull with dental acrylic (Kemdent) and stainless-steel screws (1.2 mm × 3 mm; MDK Fasteners). Nonprotruding double stylets (33 gauge; Plastics One, Bilaney Consultants) were inserted into the guide cannulae and a dust cap was secured on top. Rats received daily prophylactic antibiotic injections (0.02 ml/100 g, s.c., Synulox containing 14% amoxicillin; Zoetis), starting on the day of surgery, for the duration of the study. Rats were allowed to recover for at least 7 d before the start of food restriction and pretraining on the operant task.

#### mPFC drug microinfusions

Muscimol and picrotoxin doses and infusion parameters were based on our previous studies, which showed that mPFC infusions of these drugs cause neural changes consistent with neural inhibition or disinhibition (including reduced or enhanced neuronal burst firing), as well as marked attentional deficits on the 5-choice serial reaction time task (Pezze et al., 2014). Rats were gently restrained to insert 33-gauge injectors (Plastics One, Bilaney Consultants) into the previously implanted guide cannulae. The injectors protruded −0.5 mm below the guides into the mPFC, resulting in the following target coordinates for the infusions: +3 mm anterior and ±0.6 mm lateral from bregma and −4 mm ventral from skull. The injector ends were connected via polyethylene tubing to two 5 μl syringes (SGE, World Precision Instruments) secured on a microinfusion pump (sp200IZ, World Precision Instruments). Prior to infusions, the tubing and syringe were backfilled with distilled water, and an air bubble was included before any drug solution was pulled up. A volume of 0.5 µl/side of either 0.9% sterile saline (vehicle), GABA-A receptor antagonist picrotoxin (300 ng/0.5 µl/side, C30H34O13, Sigma-Aldrich) in sterile saline, or agonist muscimol (62.5 ng/0.5 µl/side, C4H6N2O2, Sigma-Aldrich) in sterile saline was administered over the span of 1 min. Movement of the air bubble within the tubing was monitored to ensure solutions had been successfully injected into the brain. Injectors remained in place for an additional 1 min to allow for absorption of the infusion bolus by the surrounding brain tissue. The injectors were then removed and the stylets replaced. Testing began 10 min after the infusion was complete. Each rat received six microinfusions into the mPFC. The number of microinfusions per rat was limited to six to reduce the risk that reactive gliosis caused by repeated microinfusions may reduce the effectiveness of the infused drug (Cunningham et al., 2008). After each infusion session, tubing and microinjectors were flushed with distilled water and cleaned with >98% ethanol.

#### Verification of cannula placements

At the end of all testing, rats were overdosed with sodium pentobarbitone (1–2 ml Euthatal; 200 mg/ml; Genus Express) and transcardially perfused with PBS followed by 4% paraformaldehyde (PFA) solution in PBS. Following extraction, brains were postfixed in 4% PFA and cut into 70 µm coronal sections using a vibratome (Leica). Sections were then mounted on slides and viewed under a light microscope, where cannula placements were verified and mapped onto coronal sections of a rat brain atlas (Paxinos and Watson, 1998). A subset of sections was cresyl violet stained and coverslipped (DPX mountant, Sigma-Aldrich) to take photographs for presentation purposes.

### Chemogenetic mPFC disinhibition

#### Viral vector injections into mPFC

We used a double-floxed Gi-coupled hM4Di DREADD fused with mCherry reporter under the control of human synapsin promoter and packed in an adeno-associated virus (AAV) vector [pssAAV-8-hSyn1-dlox-hM4D(Gi)_mCherry(rev)-dlox-WPRE-hGHp(A), https://www.addgene.org/44362/]. For injections, we used a solution of the DREADD-containing viral vector, serotype 8 (CNS tropism), in 1× PBS, pH 7.4 (including 1 mM MgCl_2_ and 2.5 mM KCl), with a physical titer of 6.3 × 10^12^ vg/ml (v84-8, Viral Vector Facility, University of Zürich). The solution was aliquoted into 12.5 µl portions into low protein binding PCR tubes and stored at −80°C until use.

We used similar stereotaxic procedures and perioperative care as for the guide cannula implants described above; because a chronic implant was not required, rats did not receive any antibiotic treatment. Coordinates and injection parameters were based on pilot studies (chapter 4 in Renström, 2024). Skull trepanations were drilled above the mPFC at +2.8 mm anterior and ±0.6 mm lateral from bregma. Double injectors (C235I-SPC, Plastics One, Bilaney Consultants) glued into double infusion guide cannulae (“mouse” model C235GS-5-1.2; Plastics One, Bilaney Consultants, with 4.0 mm projection) were used to inject the vector. The injectors were held by a cannula holder attached to the stereotaxic frame. The ends of the injectors were attached to two 5 μl syringes (SGE) via PE50 tubing (Plastics One, Bilaney Consultants), and syringes were secured on a microinfusion pump (SP200IZ syringe pump, World Precision Instruments). Prior to injections, the tubing and syringe were backfilled with distilled water, and a small air bubble was pulled into the tubing before any viral solution was pulled up. The injector tips were slowly lowered through the trepanations to −4.0 mm from the skull surface and then pulled back to the target dorsoventral coordinate of −3.8 mm, to create a small “pocket” for the injection bolus. Rats received 1.0 µl of the AAV solution bilaterally at a rate of 0.1 µl/min. Following injections, the injectors were left in place for 10 min to allow for absorption of the injection bolus. After the injectors had been withdrawn, the incision was sutured up. Rats were held for 28 d before any further procedures were conducted, to allow for strong DREADD expression (Smith et al., 2016).

#### CNO injections

We used systemic injection of CNO for DREADD activation. Previous studies have typically used 5–10 mg/kg of freebase CNO to activate DREADDs (Smith et al., 2016). For the DREADD activation in the current experiment, we used CNO-2HCl (synthesized in-house, Boehringer Ingelheim), a water-soluble CNO salt. Sterile saline (0.9%) was used as vehicle, and all systemic injections were intraperitoneal (i.p.), in a volume of 1 ml/kg. We started with an initial dose of 6 mg/kg of CNO-2HCl, which corresponds to 5 mg/kg of freebase CNO (molecular weight of CNO-2HCl compared with CNO: 415 vs 342 g/mol, a factor of 1.2). At 6.0 mg/kg, CNO-2HCl produced marked in vivo electrophysiological changes under anesthesia, in the mPFC of Vgat-Cre rats expressing the hM4Di DREADD in mPFC GABA neurons (Fig. S1). However, testing the effects of 6 mg/kg CNO-2HCl on reversal learning performance in Vgat-Cre rats expressing hM4Di in mPFC GABA neurons revealed that this dose had substantial side effects, with rats not pressing the operant levers. We, therefore, tested lower doses of 1.5, 3.0, and 4.5 mg/kg CNO-2HCl. Within the Results section, we focus on the results obtained with the 4.5 mg/kg dose. See below for additional details on the dose selection (Reversal learning experiments, Impact of mPFC muscimol and picrotoxin microinfusion and of mPFC chemogenetic disinhibition on late reversal learning performance).

#### Verification of DREADD expression

At the end of all testing, rats were overdosed and transcardially perfused as described above, Verification of cannula placements. Following extraction, brains were postfixed in 4% PFA and cut into 50 µm coronal sections using a vibratome (Leica). Sections were then mounted on slides (DPX mountant, Sigma-Aldrich) and scanned under a fluorescence microscope (Zeiss AxioScan Z1 scanner; Carl Zeiss), to map expression of the mCherry reporter that was included with the DREADD. DREADD spread was visualized using Affinity Designer (version 1.10.4, Serif). Scans showing mCherry signal were superimposed onto a digital template of the Paxinos and Watson (1998) rat atlas. Due to variability in brain shape and size, scans were manipulated to best fit the template at the approximate distance from bregma. mCherry signal was traced by hand, and opacity of the regions was reduced to 25% such that overlapping regions appeared darker.

### Reversal learning experiments

For the three reversal learning experiments, we used a food-reinforced two-lever reversal task adapted from protocols by Brady and Floresco (2015), where rats first learnt a spatial discrimination (SD), i.e., that either presses on the left or right lever were rewarded by a sucrose pellet, before undergoing serial reversals, i.e., Reversal 1, Reversal 2, etc. (R1, R2, etc.), of the discrimination on subsequent days. In the following, we first outline the general task procedures before describing the design of Reversal experiments 1–3.

#### Food-reinforced two-lever reversal task

The pretraining and testing protocols were adapted from our previous studies (Gonçalves et al., 2023) and based on original protocols by Brady and Floresco (2015).

##### Apparatus

All reversal testing was conducted in eight individual operant boxes (30.5 cm × 24.1 cm × 21.0 cm; Med Associates), equipped with a house light (40 lux), an extractor fan, two retractable response levers either side of a circular dish, into which sucrose reward pellets (5TUL-45 mg, TestDiet, OCB Systems) were dispensed, as well as two LED cue lights (40 lux each), one above each lever. The LED cue lights above the levers were illuminated pseudorandomly throughout all sessions but were not relevant to the correct lever choice (which was always determined by lever position, left or right). Each rat was assigned to an operant chamber, where it underwent all operant pretraining and test sessions. Chambers were cleaned with 20% ethanol between different rats. The stimuli presented, lever operation, and data collection were controlled via an interface with the computer and using custom software (MED-PC software, Med Associates).

##### Pretraining

One day before pretraining, rats were habituated to the apparatus by placing them in the operant box for 15 min, with exterior doors open and no levers extended. Pretraining started with several days of single-lever-press training sessions (one session per day), during which one of the two levers was extended for a fixed 30 min period, with each lever press rewarded by one sucrose pellet (5TUL-45 mg, TestDiet, OCB Systems). The initial location (left or right) of the lever was counterbalanced across rats. Lever location was switched once at least 50 responses were made in one session. At the beginning of the first lever-press session, two pellets were placed in the dish and crushed pellets on top of the extended lever to promote rats’ engagement with the apparatus. This was repeated on Day 2 if rats did not readily press the lever on the previous day (<10 presses in the first 10 min). During these sessions, rats could press levers without restriction, allowing for unlimited rewards. This stage was completed after the criterion of 50 responses was met for both levers.

Lever-press training was followed by a minimum of 5 d of 90-trial retractable-lever training sessions (one session per day), where rats had to press the extended lever within the 10 s response window, after which the lever would be retracted. Both levers were presented in a pseudorandom order, but the same lever would not be extended more than two times in a row. Each lever press was rewarded by one sucrose pellet. At the end of this stage, rats were expected to make five or fewer omissions during a 90-trial session. A total of 51/64 rats achieved this criterion by the last day. All rats had achieved several consecutive days of <5 omissions prior to the last day, with all rats making an average of 3.67 ± 0.58 (*x̄* ± SEM) omissions by the final day, and thus all rats progressed to the next stage.

On the final day of pretraining, immediately after the last 90-trial session, the side preference of each rat was determined using the procedure reported by Brady and Floresco (2015). (1) Rats were tested on seven “main-trials,” on which the rat's choice (left or right lever) was recorded, and rats received food reward regardless of which lever they pressed. The rat's side preference was defined as the side of the lever that the rat chose to start on at least four of the seven main-trials. (2) Each main-trial was followed by a variable number of “sub-trials,” on which the rat had to select the lever opposite to that chosen on the preceding main-trial to obtain a reward (i.e., left lever on main-trial followed by right lever on sub-trial, or right lever on main-trial followed by left lever on sub-trial). Sub-trial responses on the same lever as on the preceding main-trial (e.g., right lever on main-trial, followed by right lever on sub-trial) were not rewarded. The purpose of the sub-trials was to counteract any side preference the rat may have. The minimal number of trials was 14 (7 main-trials + 7 sub-trials), but rats with a stronger side bias often required additional sub-trials to progress, as a correct press on the opposite lever was required to advance to the next main-trial.

##### Spatial discrimination testing

Testing began with a spatial discrimination (SD) task, where rats were rewarded for pressing the lever opposite to their side preference established on the previous day. Trials began every 20 s, with a 10 s response window. On each trial, both levers were presented, but only the one opposite to the rats’ side bias was rewarded. Each correct response was rewarded with one sucrose pellet. Sessions were terminated once rats had reached a criterion of 10 consecutive correct responses. If the criterion was not reached within 200 trials, rats were retested with the same lever the next day. In the present study, all rats reached criterion within a maximum of two SD sessions.

##### Reversal testing

The day after completion of the SD stage, reversal testing began. As during SD testing, trials began every 20 s, with a 10 s response window. On each trial, both levers were presented, but only one was rewarded. In all three reversal experiments, each reversal stage (R1, R2, etc.) started with 20 reminder trials, where the same lever response was rewarded as on the previous day. The purpose of reminder trials was to assess if retrieval/expression of the rule learnt on the previous day was affected by the prefrontal manipulations and, thereby, to aid interpretation of any effects of these manipulations on reversal learning (Brady and Floresco, 2015). The reversal occurred after trial 20, when the correct and incorrect levers were reversed, such that the lever opposite to the one rewarded on the previous day and during the reminder trials was now the correct lever and rewarded. A reversal session ended when rats reached a criterion of 10 consecutive correct responses or after a maximum of 200 reversal trials.

#### Design of Reversal experiments 1–3

##### Early versus late reversal learning

Reversal experiment 1 was designed to study the impact of mPFC manipulations on “early” reversals, when rats were experiencing reversals for the first few times and were learning that reward contingencies can change, thereby improving their reversal performance from one reversal to the next. In contrast, Reversal experiments 2 and 3 were designed to study the impact of mPFC manipulations on “late” reversals, when rats were proficient at reversals and reversal performance no longer improved markedly from one reversal to the next.

Our pilot studies showed that rats “learned to reverse,” i.e., markedly improved their reversal performance across the first two to four reversals, but that reversal performance was relatively stable from R5 onward (chapter 2 in Renström, 2024). Therefore, Reversal experiment 1, examining the impact of mPFC manipulations on “early” reversals, when rats were “learning to reverse,” focused on the SD stage and the first few reversals up to R5; all rats completed R1–3, so we limited our analysis to these reversal stages. Reversal experiments 2 and 3, examining the impact of mPFC manipulations on “late” reversals, when rats were proficient at reversals, focused on reversal 5 onward.

##### Reversal experiment 1: impact of mPFC muscimol and picrotoxin microinfusion on early reversal learning performance

We examined the impact of mPFC muscimol and picrotoxin on early reversals using a between-subjects design. A between-subjects design was required to examine how the different mPFC infusions affect the changes in performance across early reversal stages, while rats learn to reverse. A within-subjects design, comparing the effect of different drugs within the same rat on different days, was unsuitable, because the marked day-to-day changes in performance at this task stage would have confounded the comparison of the effects of different drug infusions on different days within the same rat.

Rats (Table 1, Cohort #1) were allocated to one of the three infusion groups—saline, muscimol, or picrotoxin—using blocked randomized allocation, with at least one rat in each cage of four being allocated to each infusion group. Rats received their allocated infusions 10 min before testing at the initial SD and at the subsequent first few reversal stages (all rats completed reversals 1–3, R1–3). If rats failed to reach the success criterion within the 200-trial limit of a daily session, they were tested on the same rule (without reminder trials) the next day, 10 min after another infusion of their allocated drug. Because we limited the number of mPFC microinfusions to six, it was not possible to test the impact of mPFC microinfusions on more than six test sessions. Within these six 200-trial test sessions, all rats managed to complete the SD stage and R1–R3 and, therefore, our analysis focused on these stages.

##### Reversal experiments 2 and 3: impact of mPFC muscimol and picrotoxin microinfusion and of mPFC chemogenetic disinhibition on late reversal learning performance

The impact of mPFC manipulations on late reversals (from R5 onward) was examined using within-subjects designs. At this stage, reversal performance no longer showed marked improvements from 1 d of reversal testing to the next (chapter 2, Renström, 2024). Therefore, a within-subjects design comparing the effect of different drug infusions on different days within the same rat was most appropriate, reducing the number of rats required. Moreover, at this stage of testing, when rats are proficient at reversal learning, they typically achieve the success criterion of 10 consecutive correct responses within 1 d, i.e., within the 200-trial limit of one single reversal session. With the exception of testing involving mPFC chemogenetic disinhibition at the high dose of CNO-2HCl (6 mg/kg), which had marked nonspecific effects, such that rats failed to engage with the task (see below), this was also the case in the present study: all rats tested during the late reversals (from R5 onward) reached criterion within the 200-trial daily session limits, regardless of the mPFC manipulation condition. To reduce the risk of carryover effects, whereby any impairment caused by an mPFC manipulation during one reversal may affect performance on the next reversal and, thereby, confound the within-subjects design, we interspersed “retention days” between reversals, similar to Boulougouris et al. (2007). On retention days, rats were tested without any mPFC manipulation, in the same way, and with the same lever rewarded, as during the reversal trials of the preceding test day, up to a criterion of 10 consecutive correct responses (all rats reached criterion within the 200-trial session limit). Retention days reinforced the lever press rule from the last reversal before testing rats on the next reversal, to ensure that all rats started each new reversal with the old rule from the previous stage well established. Before the impact of mPFC manipulations on late reversals was tested, rats were trained on SD and R1–R4, with interleaved retention days, to establish stable reversal performance. Then, from R5 onward, we examined the impact of mPFC manipulations on late reversal performance within subjects.

In Reversal experiment 2, we compared the impact of mPFC saline, muscimol, and picrotoxin infusions across R5–R10. Each rat (Table 1, Cohort #2) received two series of all three different mPFC infusions across R5–R10; Infusion series 1 consisted of infusions applied 10 min before R5–7 and Infusion series 2 consisted of infusions applied 10 min before R8–10; within each infusion series, the testing order of the three infusion conditions was counterbalanced, using a Latin-square design.

In Reversal experiment 3, we compared the impact of chemogenetic mPFC disinhibition by CNO-2HCl injection with the effect of saline injection in Vgat-Cre rats expressing hM4Di in mPFC GABA neurons from R5 onward; testing order of saline and CNO-2HCl injections was counterbalanced using a Latin-square design (Table 1, Cohort #4). We started by comparing the impact of 6 mg/kg of CNO-2HCl and saline injections across R5 and R6. Chemogenetic mPFC disinhibition with 6 mg/kg of CNO-2HCl had substantial side effects: rats were very sensitive to handling and hardly pressed the levers during the 90 min test session, making reversal testing impossible at this high dose. We, therefore, tested lower doses of CNO-2HCl. A comparison of the impact of 1.5 and 3.0 mg/kg, and saline, across R7 and R9, did not reveal any gross side effects, but also no significant impairments in whole-session measures of reversal performance, although a Bayesian trial-by-trial strategy analysis revealed a dose-dependent reduction in win-stay probability around reversal (Fig. S2; for more information on whole-session performance measures and the Bayesian strategy analysis, see below, Performance measures). We, therefore, increased the dose to 4.5 mg/kg and compared this with saline at R10 and R11, which induced marked reversal impairments without inducing nonspecific side effects. Therefore, this report will focus on the 4.5 mg/kg dose in the Results section.

Finally, to verify that CNO-2HCl did not affect serial-reversal learning independent of DREADD activation, e.g., due to known back metabolism into clozapine (Jendryka et al., 2019), we compared the impact of systemic saline vehicle and 4.5 mg/kg CNO-2HCl injections on serial reversals in wild-type Lister hooded rats (Table 1, Cohort #5). These rats had undergone serial-reversal testing up to R10, for the purpose of another behavioral study. Then, at R11 and R12, we compared behavior following intraperitoneal injection of 4.5 mg/kg CNO-2HCl or saline in a cross-over within-subjects design.

##### Sample sizes

Our starting sample sizes were *n* = 16 rats per infusion group in Reversal experiment 1 (between-subjects design) and *n* = 12–16 rats in Reversal experiments 2 and 3 (within-subjects designs). These sample sizes were chosen to give a comparable power of 80% for pairwise comparisons (between-subjects: two-tailed independent *t* tests, *p* < 0.05; within-subjects: two-tailed paired *t* tests, *p* < 0.05), to detect differences between infusion groups/conditions that correspond to an effect size of Cohen's *d* of ∼1 (G*Power; Faul et al., 2009). The final sample sizes, following exclusions for various reasons, were *n* = 14–15 rats per group in Reversal experiment 1, *n* = 13 in Reversal experiment 2, and *n* = 12 in Reversal experiment 3 and *n* = 16 for the CNO control experiment (Table 1, Cohorts #1, 2, 4, and 5).

##### Blinding

In all reversal learning experiments, the main experimenter (J.G.R.) was blinded with respect to the manipulation groups and conditions. Blinding was only broken after data analysis (although the identity of the picrotoxin group/condition and the 6 mg/kg CNO-2HCl condition was evident from the increased omissions).

#### Performance measures

##### Whole-session measures

The main measure of operant task performance was “responses to criterion” (RTC), i.e., the number of total trials a rat required to reach and complete the success criterion of 10 consecutive correct responses, excluding omitted trials when the rat did not respond within the 10 s response window. For the reminder trials, which continued even if rats made 10 consecutive correct responses, “percentage correct” was used, which refers to the percentage of correct reminder-trial responses divided by the total number of reminder-trial responses, excluding omissions. For the analysis of errors during reversal sessions, previous studies used different approaches to differentiate between “perseverative errors,” which reflect difficulty in abandoning the previously learned rule, and “regressive errors,” which occur when the old rule has been abandoned but performance slips back due to errors in performing the new rule (Deuel et al., 1971; Floresco et al., 2006; Brady and Floresco, 2015; Dalton et al., 2016; Dhawan et al., 2019). We compared several different approaches, and all yielded similar conclusions. Here, we only report the outcome of the approach outlined in Brady and Floresco (2015). We counted errors as perseverative errors until a threshold of <10 total errors in a block of 16 responses was reached. After this, all errors were counted as regressive. Additionally, we analyzed omissions (i.e., trials when rats did not press a lever within the 10 s response window), as well as response latencies for correct and incorrect responses. Performance measures during SD and reversal trials were analyzed separately from the performance measures during the reminder trials at the beginning of each reversal stage.

##### Trial-by-trial Bayesian strategy analysis

Complementing the classical performance measures outlined above, we also used a trial-by-trial Bayesian strategy analysis (Maggi et al., 2024). This analysis estimates the probability of a rat applying a particular response strategy on any given trial based on evidence collected up to this trial. Each strategy (e.g., lose-shift, win-stay) was estimated independently from each other based on a set of input parameters. These parameters included the choice (i.e., right or left lever) and the outcomes (i.e., correct/rewarded or incorrect/unrewarded) of the current and preceding responses. For example, a “lose-shift” strategy is defined as an incorrect/unrewarded response (“lose”), followed by a response “shift” to the opposite lever on the following trial, whereas a “win-stay” strategy is defined as a correct/rewarded response (“win”), followed by reselection of the same lever (“stay”) on the following trial. Estimating the lose-shift/win-stay strategy probabilities, therefore, requires information about the choice and outcome on trial *t* and the choice on trial *t* + 1. Briefly, the Bayesian strategy analysis uses the Bayes rule to estimate the probability of a given strategy *i* being applied by the subject based on the history of choices up to the current trial *t*, *P*(strategy*_i_*(*t*)|choices(1:*t*)). This posterior probability is updated based on the likelihood (how well the choices match the strategy) and the prior (initial probability estimate of using that strategy). The initial estimation of the prior at trial 0 was based on the uniform distribution. Maggi et al. (2024) showed that the choice of prior (whether based on the uniform or Jeffrey's distribution) has minimal impact on the estimated strategy probabilities after just a few trials because the actual data quickly dominates over the initial prior assumptions. At subsequent estimations, probabilities at trial *t − 1* were used as priors for posterior estimations at trial *t*. The model also included a forgetting rate parameter, which was not modified from our previous study and progressively reduced the influence of past evidence while giving more weight to recent choices in the probability calculations; moreover, if no evidence was available for or against a given strategy on a particular trial, the strategy's probability remained the same as on the previous trial (Maggi et al., 2024). To establish a robust estimation of strategy probabilities in the study of early reversals, we used the SD stage to accumulate evidence for each strategy before statistically comparing the impact of mPFC manipulations on probabilities at all stages from R1 onward. In the study of late reversals, evidence for each strategy was accumulated across the SD stage and R1–R4 before comparing the impact of mPFC manipulations on probabilities from R5 onward.

We investigated strategies around reversal (i.e., responses immediately preceding and following the rule reversal) and preceding learning (i.e., responses preceding the 10 consecutive correct responses that we defined as the criterion of successful learning and that ended the test sessions). These time points were of interest, as response strategies around rule reversal reveal how the rats respond to the rule change, whereas strategies preceding learning reveal how rats ultimately learn the new rule. The number of responses included in our Bayesian strategy analysis was limited by the number of responses that were completed by all rats across all sessions. For the mPFC microinfusion studies, strategy analysis prior to rule reversal was limited to six reminder-trial responses. We kept this consistent for the analysis of the chemogenetic study, although in the chemogenetic study more reminder trials would have been available for strategy analysis because chemogenetic disinhibition caused less omissions. For both microinfusion studies, 16 responses following reversal were included (i.e., overall 22 responses around rule reversal, 6 before and 16 after reversal), but because some rats in the chemogenetic study reached criterion in only 10 responses, we could only include 10 responses after reversal in that analysis (i.e., overall 16 responses around rule reversal, 6 before and 10 after reversal).

As the last 11 responses of every session were identical (one incorrect response preceding the success criterion of ten correct responses), our analysis of strategies preceding learning focused on the last five responses before the last incorrect response in that session. In the chemogenetic study, due to several rats achieving criterion within 10 responses after reversal, we could not analyze strategies around learning, because in these cases the defined point of learning (first of the 10 successive correct responses to reach criterion) was identical with the point of rule reversal and, therefore, there were no responses prior to learning after rule reversal.

We examined the following strategies:

1. Strategies that reflect if rats follow the task rule—“Go-previous” strategy around reversal, and its complementary strategy “go-new” around learning: The go-previous strategy involves the rat following the spatial rule that was correct prior to rule change (i.e., go-right or go-left), whereas the go-new strategy involves the rat applying the correct choice strategy after the reversal. Go-previous and go-new strategies are mutually exclusive, i.e., their probabilities add up to *p* = 1, whereby as one increases the other one decreases, and vice versa. We focused on go-previous around reversal and go-new around learning, because these provide information about how flexibly rats moved away from the previously learned rule and how quickly they learned the new rule, respectively.
2. Complementary strategies: Win-stay and lose-shift strategies are both task-appropriate strategies that would support correct responding, because the rat continues to press the correct lever or “shifts” to the other lever after an incorrect choice, respectively. In addition, to capture alternative approaches that rats may use to adjust to the reversal, we also examined strategies that were task inappropriate, such as cue-light-based strategies, and situational strategies that are neither directly appropriate nor inappropriate, such as a “sticky” strategy, which involved “sticking” with the same response regardless of outcome. We only report these probabilities if they varied substantially from chance (*p* = 0.5). Finally, for all the strategies above, there is a strategy that is mutually exclusive (go-previous and go-new; win-stay and win-shift; lose-shift and lose-stay; sticky and alternating). Because the probability of one of these two mutually exclusive strategies is implied by the probability of the other, we only analyzed the probabilities of the dominant strategy (i.e., *p* > 0.5) out of these pairs of mutually exclusive strategies.

### Validation of the chemogenetic disinhibition method: histological, in vivo electrophysiological and behavioral characterization of hM4Di DREADD expression and functionality in the mPFC

#### Histological analysis of the spread, penetrance, and specificity of hM4Di expression

A cohort of Vgat-Cre rats (*n* = 5; Table 1, Cohort #3) was used to confirm suitable DREADD expression across the mPFC. More specifically, we examined three aspects of DREADD expression: (1) the extent of DREADD spread across the mPFC and to adjacent non-mPFC regions; (2) DREADD penetrance (i.e., which proportion of GABAergic neurons expressed the DREADD); and (3) cell-type specificity of the DREADD (i.e., if DREADD expression was largely limited to GABAergic neurons). For this purpose, rats were overdosed with sodium pentobarbitone (1–2 ml Euthatal; sodium pentobarbitone, 200 mg/ml; Genus Express), 28 d following mPFC viral vector injection, and transcardially perfused with 160 ml of freshly prepared PBS followed by 160 ml PFA (4%). After extraction, brains were postfixed in 4% PFA overnight at +4°C, transferred into sucrose solution (30%) at +4°C, and shipped to facilities at Boehringer Ingelheim, Biberach, Germany, where they were cut into 50 µm coronal sections on a sliding microtome (Microm HM450, Thermo Fisher Scientific) with integrated freezer platform (BFS-3MP, Physitemp), and immediately placed into wells (24-well, Nunclon Delta Surface; Thermo Fisher Scientific) filled with 2 ml PBS and 0.01% thimerosal. Brain sections were stored at +4°C until further processing. For histological verification of DREADD expression spread (1), due to the endogenous fluorescence of the mCherry reporter, no additional staining was required, so slices were mounted using 1–2 drops of ProLong Diamond Antifade Mountant containing DAPI (Invitrogen) and coverslipped, before being scanned on a florescence microscope (Zeiss AxioScan Z1 scanner; Carl Zeiss) using a 20× objective (0.22 µm/px) controlled by ZEN software (Carl Zeiss) equipped with a Zeiss Colibri 7 LED light source (Carl Zeiss). To quantify DREADD penetrance (2) and cell-type specificity (3), we used immunohistochemical staining procedures. Blocking solution [1% bovine serum albumin (BSA), 0.3% Triton X-100, 10% normal goat serum (NGS; diluted in PBS)] was applied to the slices and incubated at room temperature for 2 h. Then, primary antibodies diluted in an antibody solution (1% BSA, 0.3% Triton X-100, 1% NGS) were added and the slices were incubated at +4°C overnight. Primary antibodies included anti-glutamate decarboxylase 67 (GAD67; rabbit, Millipore, catalog #MAB5406) labeling GAD67, a key synthesis protein of GABA, anti-calmodulin dependent protein kinase II alpha (CaMKIIa, rabbit, Novus Biologicals, catalog #NBP2-46821) labeling glutamatergic neurons, anti-choline acetyltransferase (ChAT, rabbit, Synaptic Systems, catalog #297013) labeling cholinergic neurons, and anti-red fluorescent protein (RFP, mouse, Synaptic Systems, catalog #390011) used to label and thus amplify endogenous mCherry signal. Following washing (45 min), via a washing solution (PBS, 0.1% Triton X-100), secondary antibodies conjugated to Alexa 488 and 568 (Thermo Fisher Scientific) were applied, and slides were incubated at room temperature for 3 h in the dark. Following another wash (60 min), 1–2 drops of ProLong Diamond Antifade Mountant containing DAPI (Invitrogen) were applied to each slide, and slides were coverslipped. Slices stained immunohistochemically were scanned on the Opera Phenix high-content confocal microscope (PerkinElmer) running Harmony high-content imaging software (PerkinElmer) using a 63× water immersion objective (0.19 μm/px). We used four slices per rat for GAD67 and one slice per rat for CaMKIIa, as the latter was predominantly a control staining to ensure no expression in excitatory cells.

DREADD spread was visualized as described above (Chemogenetic mPFC disinhibition, Verification of DREADD expression). For cell classification and quantification, scans of the immunohistochemically stained brain sections were exported into HALO (Indica Labs) running the Multiplex FISH module. Parameters for automated cell classification were determined by examination of unambiguously GAD67+, mCherry+, ChAT+, and CaMKIIa+ cells. Subsequently, each slice was examined for potential false-positive cases of cell classification, and the inclusion threshold was adjusted to best account for unambiguously positive cells, while minimizing the inclusion of false-positive cases. Finally, each slice was examined once more to confirm parameters were suitable before automated cell counting was started.

#### In vivo electrophysiology

We used in vivo electrophysiological recordings under isoflurane anesthesia to examine if chemogenetic mPFC disinhibition would cause numerical changes in LFP and multiunit measures of mPFC neural activity similar to those we had previously observed following mPFC picrotoxin (Pezze et al., 2014). First, electrophysiological recordings were conducted in the same Vgat-Cre rats (*n* = 5) that we used for the initial histological characterization of DREADD expression (see above; Table 1, Cohort #3), using 6.0 mg/kg CNO-2HCl. Second, after our reversal study indicated that 6.0 mg/kg caused nonspecific behavioral effects, whereas 4.5 mg/kg CNO-2HCl specifically disrupted late reversal learning, we reexamined the effects of 4.5 mg/kg in a subset of rats that contributed to the reversal study after all behavioral testing was completed (*n* = 5; Table 1, Cohort #4). In the Results section, we focus on the 4.5 mg/kg dose.

Surgical and recording methods were as described in detail previously (Pezze et al., 2014). A four-channel microwire array (four 50 µm Teflon-coated stainless-steel electrodes, spaced 0.25 mm apart and arranged in one row spanning ∼1 mm, with a stainless-steel ground wire; MicroProbes for Life Science) was slowly lowered into the brain, with the array arranged parallel to the midline and the center of the array aimed at the following target coordinates in the right mPFC: +3.2 mm anterior and +0.6 mm to the right from bregma and −4.0 mm ventral from the skull.

Positioning of the electrodes at the target coordinates was followed by a stabilization period of at least 30 min, during which anesthesia was adjusted to a stable level (50 ± 10 breaths per min, ∼1.5–2% isoflurane). Following a 30 min recording of baseline neural activity without any injection, rats received an intraperitoneal injection of saline (0.9%; 1 ml/kg), and neural activity was recorded for another 30 min period; then rats received an intraperitoneal injection of CNO-2HCl and neural activity was recorded for 3 h following the injection. For the intraperitoneal injections, the rat's right hindlimb was gently raised to inject into the lower abdomen; the hindlimb was gently lowered again, and the start and end times of the injection were noted to accurately identify the start and end points of the pre- and postinjection periods.

Following the post-CNO-2HCl period during electrophysiological recordings, a current (1 mA, 10 s) was passed through the first and fourth microwires to create an electrolytic lesion at the tip of the electrode and mark its position. Following this marking, the electrode assembly was removed from the brain. Subsequently, pentobarbital was administered for a deeper anesthesia, rats were transcardially perfused as above, and brains were extracted and cut into 70 µm coronal sections, which were used to visualize spread of DREADD expression as described above and to map locations of marked electrode tips onto coronal sections of the rat brain atlas (Paxinos and Watson, 1998) using a light microscope (Leica DM 1000; Leica Microsystems).

Multiunit and LFP data were recorded using a Plexon multichannel acquisition processor (MAP) system and analyzed using NeuroExplorer (version 3.2.2.6, Nex Technologies), as described in detail in our previous study (Pezze et al., 2014).

#### Open-field measurement of locomotor activity

Using the same rats we used to examine the impact of mPFC chemogenetic disinhibition on late reversals (Table 1, Cohort #4, *n* = 12), we investigated the impact of mPFC chemogenetic disinhibition on locomotor activity in an open field, after all reversal testing had been completed. The open-field locomotor measurements were completed as described previously in our studies of mPFC disinhibition by picrotoxin (Pezze et al., 2014). In brief, locomotor measurements were conducted in 12 clear Perspex chambers (39.5 cm × 23.5 cm × 24.5 cm), surrounded by a two-level frame containing 12 photobeams (4 × 8 configuration; Photobeam Activity System, San Diego Instruments), and placed in a dimly illuminated room (50–70 lux). Two consecutive breaks of adjacent beams within the lower level of photobeams generated one locomotor count. Total locomotor counts were calculated for each 10 min block of testing. Sessions started automatically once rats were placed in the chambers and a first beam break was recorded. The effect of saline and 4.5 mg/kg CNO-2HCl injections on locomotor activity was compared in Vgat-Cre rats expressing the inhibitory DREADD in GABAergic neurons in the mPFC, using a within-subject design. Activity was measured across five successive days: Day 1 was a baseline/habituation day, on which activity was recorded for a 60 min period without any manipulations. Days 2 and 4 were “injection days”, beginning with 30 min of baseline recordings, after which rats received intraperitoneal injections of either CNO-2HCl or saline. Following injections, rats were placed back in their chambers and activity was recorded for 60 min. Testing order of saline and CNO-2HCl injections was counterbalanced across rats. Days 3 and 5 were rebaseline days on which activity was recorded for a 60 min period, without any injection (as on Day 1); rebaseline days served to detect any carryover effects from the preceding injection days. The main experimenter (J.G.R.) was blinded with respect to the injection conditions, and blinding was only broken after data analysis.

Locomotor counts were binned in 10 min blocks, giving six 10 min blocks for each baseline day, and nine 10 min blocks for each injection day (three preinjection and six postinjection blocks). These values were subsequently averaged across rats for each 10 min block.

### Statistical analysis

All statistical analyses were carried out in JASP (version 0.19.3), and all graphs were made in GraphPad Prism (version 10.4.2). The accepted level of significance was *p* < 0.05 for all analyses.

For the study examining the impact of mPFC muscimol or picrotoxin on early reversals, whole-session performance measures were analyzed by ANOVA, using infusion group (saline, picrotoxin, muscimol) as a between-subjects factor and task stage (SD, R1–3) as a within-subjects factor. For the study examining the impact of mPFC muscimol or picrotoxin on late reversals, whole-session performance measures were analyzed by ANOVA using infusion condition (saline, picrotoxin, muscimol) and infusion series (1 or 2) as within-subject factors. For the studies examining the impact of chemogenetic mPFC disinhibition on late reversals and locomotor activity, systemic injection (CNO-2HCl or saline) was used as a within-subjects factor, and a 10 min block was used as an additional within-subjects factor for the locomotor data. For all reversal learning studies, trial-by-trial strategy probabilities were analyzed by ANOVA, as outlined for the whole-session performance measures, and response number was used as an additional within-subjects factor (we refer to “responses,” instead of “trials,” due to the removal of omission trials in these analyses). Any significant interactions observed were further examined by simple main effects analyses, and simple main effects were further analyzed by pairwise comparisons, using Fisher's LSD test. Where a significant interaction was observed between multiple factors, the main effects of these factors were not reported, as these were obsolete given the interactions involving these factors. A statistical comparison of LFP and multiunit measurements following CNO-2HCl or saline injection was not originally planned. However, to provide statistical support for the observed numerical changes in these measures, we averaged values across the six 5 min blocks following saline and CNO-2HCl injections, respectively, and compared these two averages within subjects; testing was one-tailed because we had directional hypotheses for the changes in LFP and multiunit measures based on our previous studies of mPFC disinhibition by picrotoxin (Pezze et al., 2014). Where the assumption of sphericity for ANOVA was violated, as indicated by a significant Mauchly's test of sphericity, Greenhouse–Geisser correction was applied to the degrees of freedom. For pairwise comparisons of the electrophysiological average values across the 30 min periods after saline and CNO-2HCl injections, we used paired *t* tests, unless the assumption of normality was violated, as indicated by a significant Shapiro–Wilk test, in which case we used nonparametric Wilcoxon signed-rank tests.

## Results

### Reversal experiment 1: mPFC functional inhibition by muscimol, but not disinhibition by picrotoxin, impaired early reversal learning

**Figure 1.**
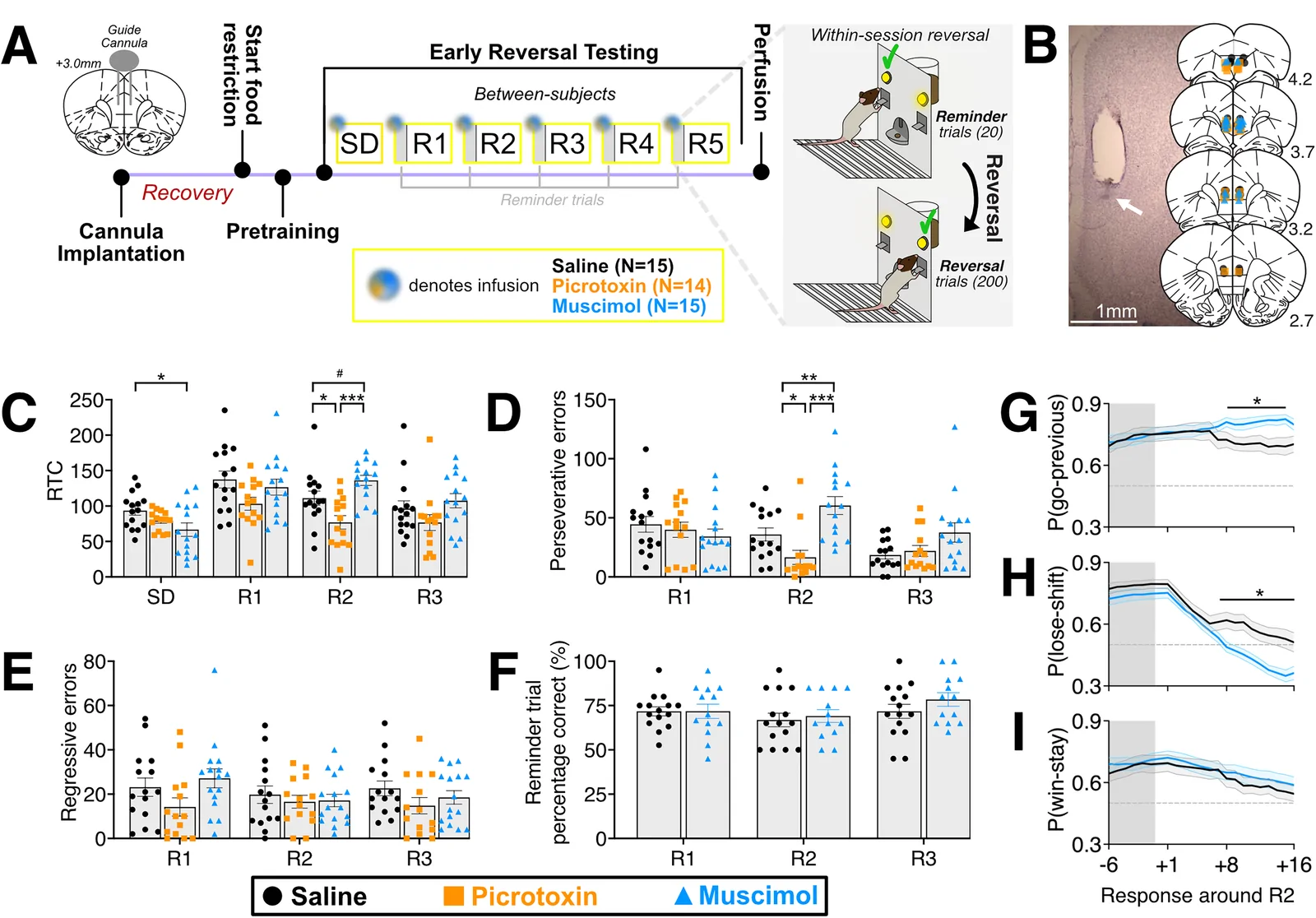
Reversal experiment 1: mPFC functional inhibition by muscimol, but not disinhibition by picrotoxin, impaired early reversal learning. ***A***, Timeline of the experiment to compare the impact of mPFC infusion of saline, muscimol, or picrotoxin (between-subjects) on early reversal learning, from cannula implantation surgery, across food restriction, pretraining, and reversal testing until transcardial perfusion. Tricolored dots indicate infusion time points 10 min before the spatial discrimination (SD) stage and before each reversal session at reversal stage 1–5 (R1–R5). The structure of the reversal sessions is illustrated: sessions started with 20 reminder trials, during which rats were tested for their expression of the “old” rule from the previous session (e.g., press the right lever), following which reward contingencies were reversed (e.g., left lever was now rewarded) and rats received a maximum of 200 reversal trials. ***B***, Cresyl violet-stained coronal brain section showing an infusion cannula placement in the prelimbic mPFC, with the tip indicated by a white arrow. Overlayed are the approximate locations of infusion cannula tips, separated by infusion group (saline, black dots; picrotoxin, orange squares; muscimol, blue triangles), shown on coronal plates adapted from the atlas by Paxinos and Watson (1998). Distance (in mm) anterior to bregma, according to the atlas, is indicated on the right. ***C–F***, RTC (***C***), perseverative errors (***D***), regressive errors (***E***), and percentage of correct responses during reminder trials (***F***) in the saline, muscimol, and picrotoxin groups across task stages (SD and R1–R3). Bar graphs show mean ± SEM, with individual values for each rat plotted to show the range of data. Significant differences and trends as revealed by post hoc pairwise comparisons are indicated as follows: *p < 0.05; **p < 0.01; ***p < 0.001; ^#^*p* = 0.051. ***G–I***, Bayesian analysis of strategies around reversal: mPFC functional inhibition by muscimol enhanced use of old strategy (go-previous) and disrupted lose-shift at R2. Probabilities (mean ± SEM) of different strategies are shown for the six reminder-trial responses (gray shading) preceding reversal and the 16 responses following reversal: go-previous (***G***), lose-shift (***H***), and win-stay (***I***). Horizontal dashed line indicates *p* = 0.5 (i.e., chance). Asterisks indicate responses where saline and muscimol significantly differed (*p* < 0.05).

First, we examined the effect of mPFC muscimol or picrotoxin infusion on early reversal learning (Fig. 1*A*). All infusion cannula tips were placed within the mPFC, in an area that corresponded approximately to +2.7 to +4.2 mm anterior to bregma in the atlas by Paxinos and Watson (1998) (Fig. 1*B*). Cannula tips were mostly placed in the prelimbic cortex, with some placements encroaching ventrally onto the infralimbic cortex. Importantly, the 0.5 µl infusion bolus will spread beyond the infusion site. If the spread of the infusion volume was isotropic, the bolus would occupy a sphere with a radius of 0.5 mm centered on the infusion site. However, the spread is likely facilitated in the dorsal direction by the cannula tracks, and so the drug spread is likely >0.5 mm from the infusion sites in the dorsal direction. Therefore, we would expect that the drug infusions would affect much of the dorsoventral extent of the mPFC, including prelimbic, infralimbic, and anterior cingulate cortex.

#### mPFC functional inhibition increased, whereas disinhibition reduced RTC during R2

Whole-session reversal performance indexed via RTC (responses to criterion) across the initial SD stage and the following first three reversals (R1–R3) is shown in Figure 1*C*. The saline group showed an initial reversal cost, with higher RTC at R1 compared with SD (mean ± SEM, 137.47 ± 11.97 and 93.6 ± 6.54, respectively), which decreased across reversals as learning progressed, consistent with our previous behavioral studies (chapter 2, Renström, 2024). A significant drug × stage interaction was observed (*F*_(6,123)_ = 3.340, *p* = 0.004, partial *η*^2^ = 0.140). At R2, mPFC functional inhibition by muscimol increased RTC, whereas mPFC disinhibition by picrotoxin reduced the reversal cost (*F*_(2,41)_ = 10.892, *p* < 0.001, partial *η*^2^ = 0.347). RTC in the muscimol group were higher compared with picrotoxin (*p* < 0.001, *d* = 1.730) and tended to be higher compared with saline (*p* = 0.051, *d* = 0.734). Additionally, RTC in the picrotoxin group were lower than in the saline group (*p* = 0.010, *d* = −0.997). Interestingly, muscimol improved SD learning (*F*_(2,41)_ = 3.655, *p* = 0.035, partial *η*^2^ = 0.151), reducing RTC compared with saline (*p* = 0.010, *d* = 0.987; all other pairwise comparisons, smallest *p* = 0.170, largest *d* = 0.519). No significant differences were observed at R1 (*F*_(2,41)_ = 2.540, *p* = 0.091, partial *η*^2^ = 0.110) or R3 (*F*_(2,41)_ = 2.060, *p* = 0.140, partial *η*^2^ = 0.091).

#### mPFC functional inhibition increased, whereas disinhibition reduced, perseverative errors during R2

To further investigate which aspects of behavior were affected by the manipulations, we next examined perseverative and regressive errors (Fig. 1*D*,*E*). For perseverative errors, we observed a significant drug × stage interaction (*F*_(4,82)_ = 4.852, *p* = 0.001, partial *η*^2^ = 0.191). There was a main effect of drug at R2 (*F*_(2,41)_ = 11.625, *p* < 0.001, partial *η*^2^ = 0.362), where mPFC muscimol increased perseverative errors compared with both saline (*p* = 0.009, *d* = 0.997) and picrotoxin (*p* < 0.001, *d* = 1.785), and mPFC picrotoxin reduced perseverative errors compared with saline (*p* = 0.040, *d* = 0.787). In addition, a trend toward a main effect of drug was observed at R3 (*F*_(2,41)_ = 3.134, *p* = 0.054, partial *η*^2^ = 0.133), but not R1 (*F* < 1, partial *η*^2^ = 0.031). Regressive errors were similar across drug groups (*F*_(2,82)_ = 2.043, *p* = 0.143, partial *η*^2^ = 0.091) and stages (main effect of stage and drug × stage interaction, *F* < 1, partial *η*^2^ = 0.043), suggesting that mPFC manipulations specifically affected perseveration without causing nonspecific learning impairments during early reversals.

#### mPFC disinhibition increased omissions, limiting reminder-trial responses

mPFC picrotoxin markedly increased the number of omissions during reminder-trial responses (*F*_(2,41)_ = 173.014, *p* < 0.001, partial *η*^2^ = 0.894), across all task stages (only a trend toward a drug × stage interaction was observed, *F*_(3.432,70.365)_ = 2.416, *p* = 0.066, partial *η*^2^ = 0.105), compared with saline (*p* < 0.001, *d* = 4.735) and muscimol (*p* < 0.001, *d* = 4.701), whereas saline and muscimol did not differ (*p* = 0.333, *d* = 0.033; Table S1). Omissions across the initial SD learning trials and the reversal trials at R1–3 were also markedly increased by picrotoxin. There was a drug × stage interaction (*F*_(3.895,79.856)_ = 4.291, *p* = 0.004, partial *η*^2^ = 0.173), with a main effect of drug infusion at every stage (smallest *F*_(2,41)_ = 19.436, *p* < 0.001, partial *η*^2^ = 0.487). Specifically, at all stages, mPFC picrotoxin increased omissions compared with both saline and muscimol (all *p* < 0.001, smallest *d* = 2.001), which did not differ from each other at any stage (smallest *p* = 0.333, *d* = 0.092). This increase in omissions by mPFC neural disinhibition is consistent with previous findings (Pezze et al., 2014; Auger et al., 2017). This marked increase in reminder-trial omissions in the picrotoxin group limited the number of reminder-trial responses (average reminder-trial responses across R1–R3 out of a maximum of 20, 3.86 ± 0.86, mean ± SEM) compared with the saline (19.78 ± 0.09) and muscimol (19.66 ± 0.11) group, limiting reinforcement of the previous rule and, thereby, likely contributing to the apparent reduction in RTC in the picrotoxin group.

#### mPFC functional inhibition did not affect correct reminder-trial responses

Due to the marked increase in reminder-trial omissions, and consequently reduced number of responses during reminder trials, the analysis of percentage correct measures for reminder-trial responses was rendered unreliable in the picrotoxin group. Therefore, the picrotoxin group was excluded from the analysis of the percentage correct values during reminder trials. Analysis of percentage correct measures limited to the muscimol and saline groups revealed that prefrontal muscimol did not affect correct responses during reminder trials compared with saline (no main effect of drug or stage, and no drug × stage interaction, largest *F*_(2,52)_ = 2.149, *p* = 0.127, partial *η*^2^ = 0.076; Fig. 1*F*).

#### mPFC disinhibition increased response latencies

Next, we examined latencies of correct and incorrect responses across reminder trials and reversal trials (Table S1). Due to the high number of omissions, only one rat receiving mPFC picrotoxin made at least one correct and one incorrect response during the 20 reminder trials at all three reversal stages (compared with all 15 rats in both the saline and muscimol groups). Therefore, we restricted the analysis of reminder-trial latencies across the three reversal stages to the saline and muscimol groups, which revealed no main effect or interaction involving drug (largest *F*_(1,28)_ = 1.773, smallest *p* = 0.194, partial *η*^2^ = 0.060). All three groups could be included in the analysis of latencies during the SD trials and the reversal trials of the three reversal stages (R1–3). Consistent with previous studies showing increased response latencies following mPFC disinhibition (Enomoto et al., 2011; Auger and Floresco, 2014; Pezze et al., 2014; Auger et al., 2017), this analysis revealed increased response latencies following mPFC picrotoxin regardless of response type and at all task stages. ANOVA revealed a significant drug × stage × response type (correct vs incorrect) interaction (*F*_(6,117)_ = 2.367, *p* = 0.034 , partial *η*^2^ = 0.108). We further analyzed this triple interaction, using a two-way ANOVA at each stage (SD, R1–3), with drug and response type as factors. This showed a main effect of drug at each stage (smallest *F*_(2,39)_ = 3.954, *p* = 0.027, partial *η*^2^ = 0.169), as well as a main effect of response type at SD, R1, and R3 (smallest *F*_(1,39)_ = 7.652, *p* = 0.009, partial *η*^2^ = 0.164). Furthermore, there was a significant drug × response type interaction at R1 (*F*_(2,39)_ = 6.364, *p* = 0.004, partial *η*^2^ = 0.246), but not the other stages (largest *F*_(2,39)_ = 1.191, *p* = 0.315, partial *η*^2^ = 0.058). Post hoc pairwise comparisons at SD, R2, and R3 revealed that, at all these stages, picrotoxin increased response latencies compared with saline and muscimol infusions (largest *p* = 0.008, *d* = 1.011), which did not differ (smallest *p* = 0.157, *d* = 0.489). Simple main effects analysis of the interaction at R1 revealed a main effect of drug on both correct and incorrect latencies (smallest *F*_(2,39)_ = 12.842, *p* < 0.001, partial *η*^2^ = 0.397). Post hoc pairwise comparisons revealed that, also at R1, picrotoxin increased both correct and incorrect response latencies compared with saline and muscimol (all *p* < 0.001, *d* = 1.513), which did not differ from each other (smallest *p* = 0.320, *d* = 0.367).

#### Bayesian analysis of strategies around reversal: mPFC functional inhibition enhanced use of the old strategy (go-previous) and disrupted lose-shift at R2

To complement classical whole-session measures of reversal performance, and to gain an insight into the underlying behavioral changes across the trials within a session, we first examined rats’ response strategies during responses around rule reversal (six reminder-trial responses and 16 reversal-trial responses), using the Bayesian trial-by-trial strategy analysis (Maggi et al., 2024). The picrotoxin group was excluded from this analysis because of the low number of reminder trials completed by this group due to the high number of omissions (see above). Analysis of the go-previous probability in the saline and muscimol groups across all reversal stages (R1–3, data not shown) revealed a significant drug × stage × response interaction (*F*_(42,1176)_ = 2.235, *p* < 0.001, partial *η*^2^ = 0.074). Further analysis of this triple interaction revealed a significant drug × response interaction at R2 (*F*_(3.340,93.078)_ = 6.456, *p* < 0.001, partial *η*^2^ = 0.187; Fig. 1*G*), whereas, at R1 and R3, there was no main effect or interaction involving drug (largest *F*_(2.461,68.910)_ = 1.350, *p* *=* 0.267, partial *η*^2^ = 0.046; data not shown). The drug × response interaction at R2 reflected that mPFC functional inhibition increased the go-previous probability compared with saline between responses 9 and 15 after reversal (smallest *F*_(1,28)_ = 5.165, *p* = 0.031, partial *η*^2^ = 0.156), with trends toward an increase at responses 8 (*F*_(1,28)_ = 3.980, *p* = 0.056, partial *η*^2^ = 0.124) and 16 (*F*_(1,28)_ = 4.090, *p* = 0.053, partial *η*^2^ = 0.127) after reversal, in line with the increased RTC and perseverative errors caused by mPFC muscimol at that stage (Fig. 1*C*,*D*).

Before rule reversal, during reminder trials, rats showed high lose-shift (Fig. 1*H*) and high win-stay (Fig. 1*I*) probabilities. The high lose-shift probabilities may partly reflect that rats made very few incorrect responses during reminder trials, and, therefore, the lose-shift probability was hardly updated by our Bayesian analysis algorithm and remained at similarly high values as at the end of the preceding reversal session. The high win-stay probabilities were consistent with the high percentage of correct responses during reminder trials. However, immediately after reversal, there was a steep decline in lose-shift behavior and a more gradual decline in win-stay behavior, similar to our previous findings following the switch from a spatial rule to a cue-based rule (Maggi et al., 2024), revealing that the rule reversal induced marked changes in win-stay and lose-shift behavior. Analysis of lose-shift probabilities across R1 to 3 (data not shown) revealed a drug × stage × response interaction (*F*_(42,1176)_ = 1.436, *p* = 0.037, partial *η*^2^ = 0.049). This triple interaction reflected that lose-shift behavior was reduced by muscimol, compared with saline, specifically at R2, between responses 8 and 16 after reversal (drug × response interaction, *F*_(1.803,50.471)_ = 3.569, *p* = 0.040, partial *η*^2^ = 0.113; main effect of drug at responses 8–16, smallest *F*_(1,28)_ = 6.795, *p* = 0.014, partial *η*^2^ = 0.195; main effect of drug for all other responses, largest *F*_(1,28)_ = 2.797*, p* = 0.106, partial *η*^2^ = 0.091; Fig. 1*H*). In contrast, at R1 and R3, there was no main effect or interaction involving drug (largest *F*_(1,28)_ = 1.250, *p* = 0.273, partial *η*^2^ = 0.043), only a main effect of response (smallest *F*_(2.094,54.390)_ = 40.771, *p* < 0.001, partial *η*^2^ = 0.593; data not shown). In contrast to lose-shift behavior, win-stay behavior was not affected by mPFC drug infusions but declined similarly following both mPFC saline and muscimol and across all reversal stages following reversal (only a main effect of response, *F*_(2.863,80.174)_ = 81.264, *p* < 0.001, partial *η*^2^ = 0.744, but no other main effects or interactions, largest *F*_(42,1176)_ = 1.289, *p* = 0.104, partial *η*^2^ = 0.044; data for R2 are shown in Fig. 1*I*).

#### Bayesian analysis of strategies preceding learning: no effect of mPFC functional inhibition

Contrasting with the group differences in strategy use around reversal, mPFC muscimol did not affect strategies leading up to the point of learning (data not shown). Analysis of go-new, lose-shift, and win-stay probabilities during responses −5 to −1 leading up to learning only revealed an effect of response for each strategy (smallest *F*_(1.223,34.246)_ = 5.288, *p* = 0.022, partial *η*^2^ = 0.159), reflecting that these task-pertinent strategies increased leading up to reaching the performance criterion, without any other main effect or interaction involving drug (largest *F*_(1.387,38.830)_ = 1.667, *p* = 0.206, partial *η*^2^ = 0.056).

### Reversal experiment 2: mPFC disinhibition by picrotoxin, but not functional inhibition by muscimol, impaired late reversal learning

**Figure 2.**
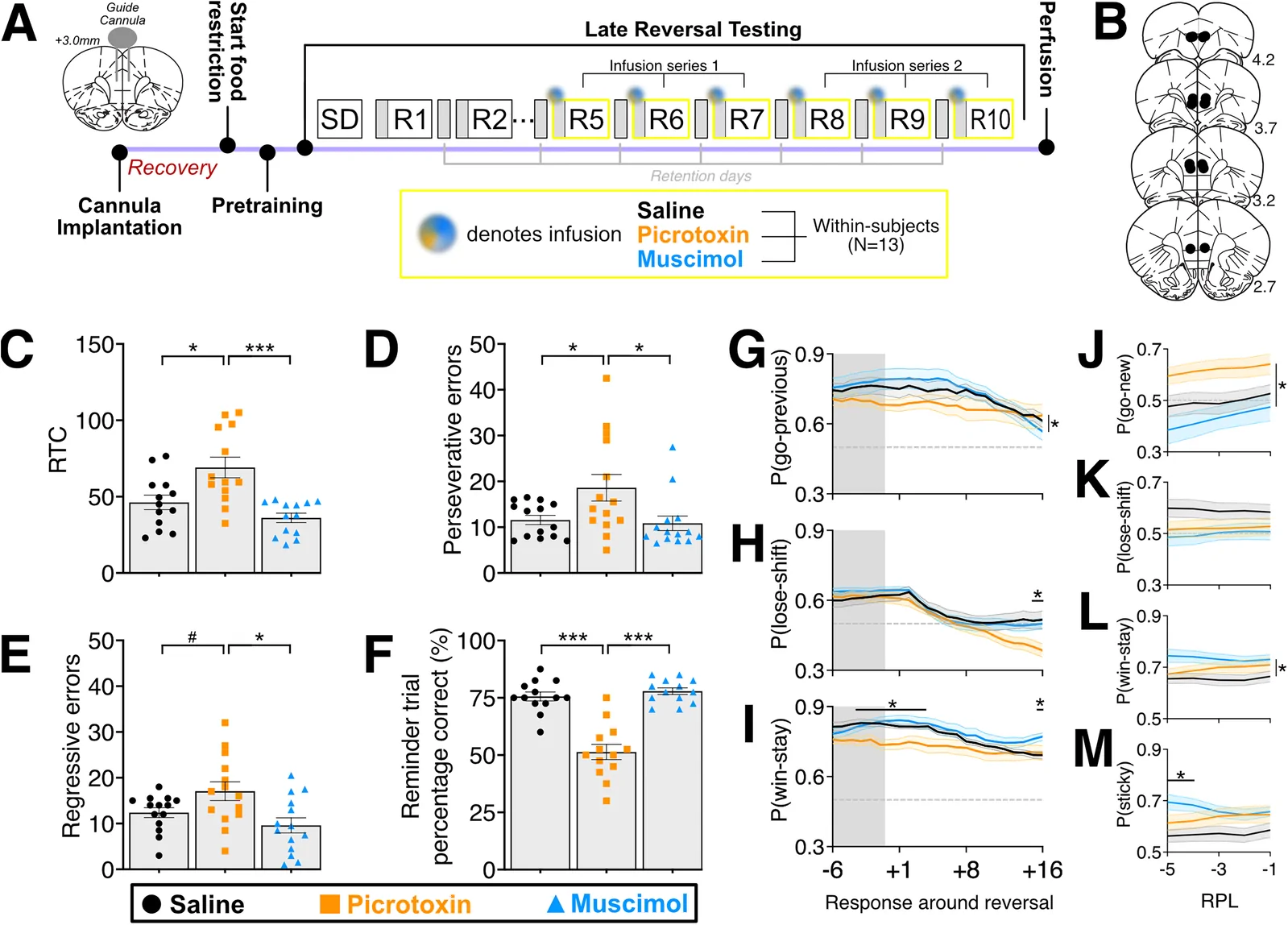
Reversal experiment 2: mPFC disinhibition by picrotoxin, but not functional inhibition by muscimol, impaired late reversal learning. ***A***, Timeline of the experiment to examine the impact of mPFC infusion of saline, muscimol or picrotoxin (within-subjects) on late reversal learning, from cannula implantation surgery, across food restriction (FR), spatial discrimination (SD), and early reversal (reversals 1–4, R1–4) training and late reversal testing (R5–R10) until transcardial perfusion. Tricolored dots indicate infusion time points 10 min before R5 to R10. Retention days were included between reversal sessions, and rats completed two infusion series, where they underwent the same series of three different infusions (saline, muscimol, and picrotoxin) twice. ***B***, Approximate locations of infusion cannula tips (black dots), shown on coronal plates adapted from the atlas by Paxinos and Watson (1998). Distance (in mm) anterior to bregma, according to the atlas, is indicated on the right. ***C–F***, RTC (***C***), perseverative errors (***D***), regressive errors (***E***), and percentage of correct responses during reminder trials (***F***) in the saline, muscimol, and picrotoxin conditions, averaged across both infusion series. Bar graphs show mean ± SEM, with individual values for each rat plotted to show the range of data. Significant differences as revealed by post hoc pairwise comparisons are indicated as follows: *p < 0.05; **p < 0.01; ***p < 0.001. ***G–I***, Probabilities (mean ± SEM) of different strategies are shown for the six reminder-trial responses (gray shading) preceding reversal and the 16 responses following reversal: go-previous (***G***), lose-shift (***H***), and win-stay (***I***). Horizontal dashed line indicates *p* = 0.5 (i.e., chance). Asterisks above data points indicate responses where groups significantly differed (*p* < 0.05) following observation of a drug × response interaction. Main effect of drug across responses, without an interaction, is indicated by a horizontal line and asterisk to the right of the data lines. ***J–M***, Probabilities (mean ± SEM) of different strategies are shown for the five responses preceding learning (RPL): go-new (***J***), lose-shift (***K***), win-stay (***L***), and sticky (***M***). Horizontal dashed line indicates *p* = 0.5 (i.e., chance). Significant differences are indicated as for ***G–I***.

Next, we tested the effect of mPFC muscimol or picrotoxin infusion on late reversal learning, when reversal performance was well established, across R5–10 (Fig. 2*A*). All infusion cannula tips were placed within the mPFC (Fig. 2*B*).

#### mPFC disinhibition increased RTC during late reversal learning

mPFC picrotoxin markedly impaired late reversal performance, reflected by higher RTC (main effect of drug, *F*_(2,24)_ = 9.603, *p* < 0.001, partial *η*^2^ = 0.445), compared with saline (*p* = 0.006, *d* = 0.979) and muscimol (*p* *=* 0.001, *d* = 1.340), which did not differ from each other (*p* = 0.266, partial *η*^2^ = 0.044; Fig. 2*C*). There was a trend toward a main effect of infusion series (*F*_(1,12)_ = 4.255, *p* = 0.058, partial *η*^2^ = 0.268), but no drug × infusion series interaction (*F* < 1, partial *η*^2^ = 0.061), which reflected that RTC tended to decline slightly from series 1 to 2 across all infusion conditions (data not shown).

#### mPFC disinhibition increased both perseverative and regressive errors

The increase in RTC by mPFC picrotoxin reflected an increase in perseverative errors (main effect of drug, *F*_(1.269,15.231)_ = 4.592, *p* = 0.041, partial *η*^2^ = 0.277) compared with saline (*p* = 0.043, *d* = 0.733) and muscimol (*p* = 0.046, *d* = 0.815; Fig. 2*D*) and in regressive errors (main effect of drug, *F*_(2,24)_ = 5.427, *p* = 0.011, partial *η*^2^ = 0.311) compared with muscimol (*p* = 0.003), with a trend toward an increase compared with saline (*p* = 0.050, *d* = 0.964; Fig. 2*E*). Errors did not differ between saline and muscimol conditions (smallest *p* = 0.175, *d* = 0.353)*.* There was no main effect of infusion series or drug × infusion series interaction (largest *F*_(1.199,14.383)_ = 2.400, *p* = 0.140, partial *η*^2^ = 0.167).

#### mPFC disinhibition impaired expression of the previous rule during reminder trials

mPFC disinhibition markedly impaired the expression of the old rule prior to rule change, as reflected by a reduced percentage of correct responses during reminder trials ([correct responses / all responses] × 100%; main effect of drug, *F*_(2,24)_ = 24.673, *p* < 0.001, partial *η*^2^ = 0.673; Fig. 2*F*). The percentage of correct reminder trials was reduced by picrotoxin compared with saline and muscimol infusions (both *p* < 0.001, smallest *d* = 1.253), which did not differ from each other (*p* *=* 0.333, *d* = 0.062). No main effect or interaction involving infusion series was observed (main effect of infusion series, *F* < 1, partial *η*^2^ = 0.059; drug × infusion series interaction, *F*_(1.191,22.939)_ = 2.778, *p* = 0.085, partial *η*^2^ = 0.188).

#### mPFC disinhibition increased omissions and response latencies

Consistent with previous studies and our early reversal learning experiment (see above), mPFC disinhibition markedly increased omissions and latencies of correct and incorrect responses across both reminder and reversal trials, whereas functional inhibition did not affect these measures (Table S2). However, compared with our early reversal learning experiment, omissions were overall lower, and the increase in omissions by mPFC picrotoxin was not as detrimental to reminder trial responding. Nevertheless, a main effect of drug was still observed for reminder-trial omissions (*F*_(1.003,12.04)_ = 5.510, *p* = 0.037, partial *η*^2^ = 0.315) and reversal-trial omissions (*F*_(1.002,12.02)_ = 24.204, *p* < 0.001, partial *η*^2^ = 0.669); in both cases, this was driven by increased omissions following mPFC picrotoxin compared with saline and muscimol (largest *p* = 0.036, *d* = 0.793), whereas saline and muscimol did not differ (both *p* = 0.333, *d* = 0.004). No main effect or interaction involving series was observed for omissions during reminder or reversal trials (largest *F*_(2,24)_ = 2.779, *p* = 0.082, partial *η*^2^ = 0.188). For reminder-trial response latencies, there was a significant drug × response type interaction (*F*_(2,22)_ = 4.317, *p* = 0.026, partial *η*^2^ = 0.282), and no other main effect or interaction involving infusion series or response type (all *F* < 1). Simple main effects analysis showed a significant effect of drug for both correct- and incorrect-response latencies (smallest *F*_(1.330,15.957)_ = 8.676, *p* = 0.006, partial *η*^2^ = 0.420). Pairwise comparisons revealed that picrotoxin increased both correct and incorrect reminder-trial response latencies compared with saline and muscimol (largest *p* = 0.027, *d* = 1.245), which did not differ (*p* = 0.139, *d* = 0.360). For reversal-trial response latencies, there was also a significant drug × response type interaction (*F*_(2,24)_ = 3.902, *p* = 0.034, partial *η*^2^ = 0.245), alongside a significant drug × infusion series interaction (*F*_(1.078,12.940)_ = 4.747, *p* = 0.046, partial *η*^2^ = 0.283). Subsequent simple main effects analyses revealed significant main effects of drug across infusion series and response types (smallest *F*_(2,24)_ = 28.624, *p* < 0.001, partial *η*^2^ = 0.662). Pairwise comparisons revealed that this effect was driven by increased response latencies following picrotoxin, compared with saline and muscimol for both series and response types (largest *p* < 0.001, *d* = 2.319); muscimol and saline did not differ regardless of infusion series or response type (all *p* = 0.333, *d* = 0.098).

#### Bayesian analysis of strategies around reversal: mPFC disinhibition reduced adherence to old strategy (go-previous), as well as win-stay and lose-shift behavior

During late reversals, rats receiving mPFC saline or muscimol infusions showed high go-previous probabilities during the six last responses before the rule change; go-previous probabilities remained high until about the seventh response after rule change when they began to decrease (Fig. 2*G*), indicating that rats abandoned the previous rule and switched to the opposite lever. This decline in go-previous behavior was evident regardless of infusion series (i.e., during R5–7 and R8–10) and differed from the pattern we observed during early reversals (R1–R3), where the go-previous probability did not decline across the first 16 responses following rule reversal (Fig. 1*G*). Following mPFC disinhibition, however, the pattern differed: the go-previous probability tended to be reduced compared with saline and muscimol infusions during the reminder trials, at or below *p* = 0.7 (in line with reduced reminder-trial accuracy; Fig. 2*F*), but hardly declined following rule reversal, such that, toward the end of the first 16 responses after reversal, the go-previous probability was similar in all three infusion conditions (Fig. 2*G*). In support of these findings, ANOVA of the go-previous probabilities revealed a drug × response interaction (*F*_(42,504)_ = 1.651, *p* = 0.008, partial *η*^2^ = 0.121) and a trend toward a drug × infusion series interaction (*F*_(2,24)_ = 2.965, *p* = 0.071, partial *η*^2^ = 0.198), but no drug × infusion series × response interaction (*F*_(42,504)_ = 1.213, *p* = 0.174, partial *η*^2^ = 0.092). Simple main effects analysis of the drug × response interaction did not indicate a significant effect of drug for any response but indicated trends toward such an effect for responses −1, +2, and +8 around reversal (smallest *F*_(2,24)_ = 2.586, *p* = 0.096, partial *η*^2^ = 0.177) and for all other responses (largest *F*_(2,24)_ = 2.542, *p* = 0.100, partial *η*^2^ = 0.175).

The time course of lose-shift behavior during repeated reversals (Fig. 2*H*) was similar to that during early reversal stages (Fig. 1*H*). Before reversal, lose-shift probability was high across all three infusion conditions, and, after reversal, there was a steep decline in this strategy, indicating a tendency to stick with the previous, but now incorrect, response for several trials. Following mPFC saline and muscimol infusions, this decline plateaued ∼5 responses after reversal, coinciding with a decline in go-previous probability (Fig. 2*G*), which reflected that rats began to switch to the opposite lever. However, following mPFC picrotoxin, lose-shift probability declined throughout all 16 responses that were analyzed following reversal, dropping below the lose-shift probabilities in the saline and muscimol conditions at responses 15 and 16. Importantly, the similar lose-shift probabilities in all three infusion conditions prior to response 15 post-reversal suggest that mPFC picrotoxin does not reduce lose-shift probabilities beyond the typical decline seen following an unexpected rule change. Rather, mPFC picrotoxin appears to impair the mechanisms underlying prompt strategy adjustment, resulting in failure to rescue appropriate lose-shift behavior over time. These findings were supported by ANOVA of lose-shift probabilities, which revealed a significant drug × response interaction (*F*_(42,504)_ = 2.250, *p* < 0.001, partial *η*^2^ = 0.158), with no further main effects or interactions (largest *F*_(42,504)_ = 1.096, *p* = 0.319, partial *η*^2^ = 0.084). Simple main effect analysis revealed a significant effect of drug at responses 15 and 16 (smallest *F*_(2,24)_ = 4.185, *p* = 0.028, partial *η*^2^ = 0.259) and a trend at responses 13 and 14 post-reversal (smallest *F*_(2,24)_ = 3.185, *p* = 0.059; all other responses: largest *F*_(2,24)_ = 2.285, *p* = 0.123, partial *η*^2^ = 0.160). Post hoc pairwise comparisons showed that picrotoxin reduced lose-shift probabilities at both responses 15 and 16 compared with saline (largest *p* = 0.043, *d* = 1.059) and muscimol (largest *p* = 0.029, *d* = 0.903), which did not differ significantly from each other (*p* = 0.333, *d* = 0.156).

The probability of win-stay behavior during late reversals remained high around reversal, contrasting with the steep decline following reversal during early reversals stages (Fig. 1*I*) but was reduced by mPFC picrotoxin compared with saline and muscimol during some of the responses around reversal (Fig. 2*I*). ANOVA of win-stay probabilities revealed a significant drug × response interaction (*F*_(42,504)_ = 2.904, *p* < 0.001, partial *η*^2^ = 0.195), with no further main effects or interactions (largest *F*_(21,252)_ = 1.196, *p* = 0.254, partial *η*^2^ = 0.091). Simple main effect analyses revealed a significant drug effect starting four responses before, and ending four responses after reversal, as well as at the 16th response after reversal (smallest *F*_(2,24)_ = 3.778, *p* *=* 0.037, partial *η*^2^ = 0.239). Additionally, there was a trend toward an effect of drug at the fifth response before and after reversal, as well as at the 15th response after reversal (smallest *F*_(2,24)_ = 2.591, *p* *=* 0.096, partial *η*^2^ = 0.178) but not drug effect at any other response (largest *F*_(2,24)_ = 2.311 *p* = 0.121, partial *η*^2^ = 0.161). Post hoc pairwise comparisons, to analyze further the simple main effects of drug at responses −4 to +4 around reversal, showed that prefrontal picrotoxin significantly reduced win-stay probability compared with saline and muscimol (largest *p* = 0.049, *d* = 0.996), which did not differ (smallest *p* = 0.260, *d* = 0.394). On the other hand, the simple main effect of drug at response 16 after reversal was driven by increased win-stay probability in the muscimol condition, compared with saline (*p* = 0.006, *d* = 1.009) and picrotoxin (*p* = 0.036, *d* = 0.896), which did not differ between each other at that stage (*p* = 0.333, *d* = 0.114).

#### Bayesian analysis of strategies preceding learning: mPFC disinhibition increased adherence to the new rule (go-new) and functional inhibition increased win-stay and sticky behavior leading up to learning

Across the five responses leading up to the point of learning, the go-new probabilities were increased by mPFC picrotoxin compared with saline and muscimol infusions and gradually increased regardless of the infusion condition (Fig. 2*J*). The increased go-new probability, alongside increased RTC (Fig. 2*C*), suggests that, following mPFC picrotoxin, rats achieved the learning “breakthrough” (i.e., 10 consecutive correct responses) at a later stage (as reflected by higher RTC) because they required prolonged accumulation of evidence for “go-new” [i.e., *p*(go-new) > 0.5] before reaching the criterion. ANOVA of go-new probabilities revealed a main effect of drug (*F*_(2,24)_ = 4.650, *p* = 0.020, partial *η*^2^ = 0.279) and of response (*F*_(1.526,18.318)_ = 16.264, *p* < 0.001, partial *η*^2^ = 0.575), reflecting a gradual increase, alongside a trend toward an effect of series (*F*_(1,12)_ = 3.718, *p* = 0.078, partial *η*^2^ = 0.237), but no interactions (largest *F*_(2.112,25.343)_ = 1.165, *p* = 0.330, partial *η*^2^ = 0.089). Post hoc pairwise comparisons showed that, following picrotoxin infusions, go-new probabilities were higher than following muscimol infusions (*p* = 0.006, *d* *=* 0.921) and tended to be higher compared with saline infusions (*p* = 0.063, *d* = 0.599), with no differences between the latter (*p* = 0.304, *d* = 0.322).

Lose-shift probabilities were relatively stable during the last five responses preceding learning and were numerically higher in the saline condition (Fig. 2*K*), but this difference was not significant, with ANOVA not revealing any main effect or interaction involving drug (largest *F*_(3.139,37.664)_ = 2.180, *p* = 0.104, partial *η*^2^ = 0.154).

However, mPFC muscimol increased the probability of win-stay behavior (Fig. 2*L*) and of sticky behavior compared with saline and picrotoxin (Fig. 2*M*). ANOVA of win-stay probabilities revealed a main effect of drug (*F*_(2,24)_ = 5.922, *p* = 0.008, partial *η*^2^ = 0.330), alongside a trend for an effect of infusion series (*F*_(1,12)_ = 4.265, *p* = 0.061, partial *η*^2^ = 0.262; all other main effects or interactions: largest *F*_(2.534,30.408)_ = 1.874, *p* = 0.162, partial *η*^2^ = 0.135). Post hoc pairwise comparisons showed that the win-stay probabilities were higher following muscimol compared with saline (*p* = 0.002, *d* = 0.725) and tended to be higher compared with picrotoxin (*p* = 0.095, *d* = 0.366). Saline and picrotoxin did not differ (*p* = 0.101, *d* = 0.359). ANOVA of sticky behavior revealed a drug × response interaction (*F*_(2.849,34.186)_ = 3.354, *p* = 0.032, partial *η*^2^ = 0.218), but no other main effects or interactions (largest *F*_(1,12)_ = 2.757, *p* = 0.123, partial *η*^2^ = 0.187). Simple main effects analysis revealed an effect of drug at responses −5 and −4 before reaching criterion (smallest *F*_(2,24)_ = 4.555, *p* = 0.021, partial *η*^2^ = 0.275) and trends at responses −3 and −2 (largest *F*_(2,24)_ = 2.902, *p* = 0.074, partial *η*^2^ = 0.195), but no drug effect at response −1 (*F*_(2.24)_ = 2.012, *p* = 0.156, partial *η*^2^ = 0.144). Post hoc pairwise comparisons indicated higher sticky probability in the muscimol condition compared with saline and picrotoxin at response −5 (largest *p* = 0.044, *d* = 0.806) and to saline (*p* = 0.006, *d* = 1.310), but not picrotoxin (*p* = 0.119, *d* = 0.623), at response −4. Saline and picrotoxin did not differ from one another at any point (smallest *p* = 0.175, *d* = 0.538). This suggests that, following mPFC muscimol, rats reached criterion during late reversal learning by using slightly different strategies than in the other two conditions.

### Reversal experiment 3: chemogenetic mPFC disinhibition impaired late reversal learning

**Figure 3.**
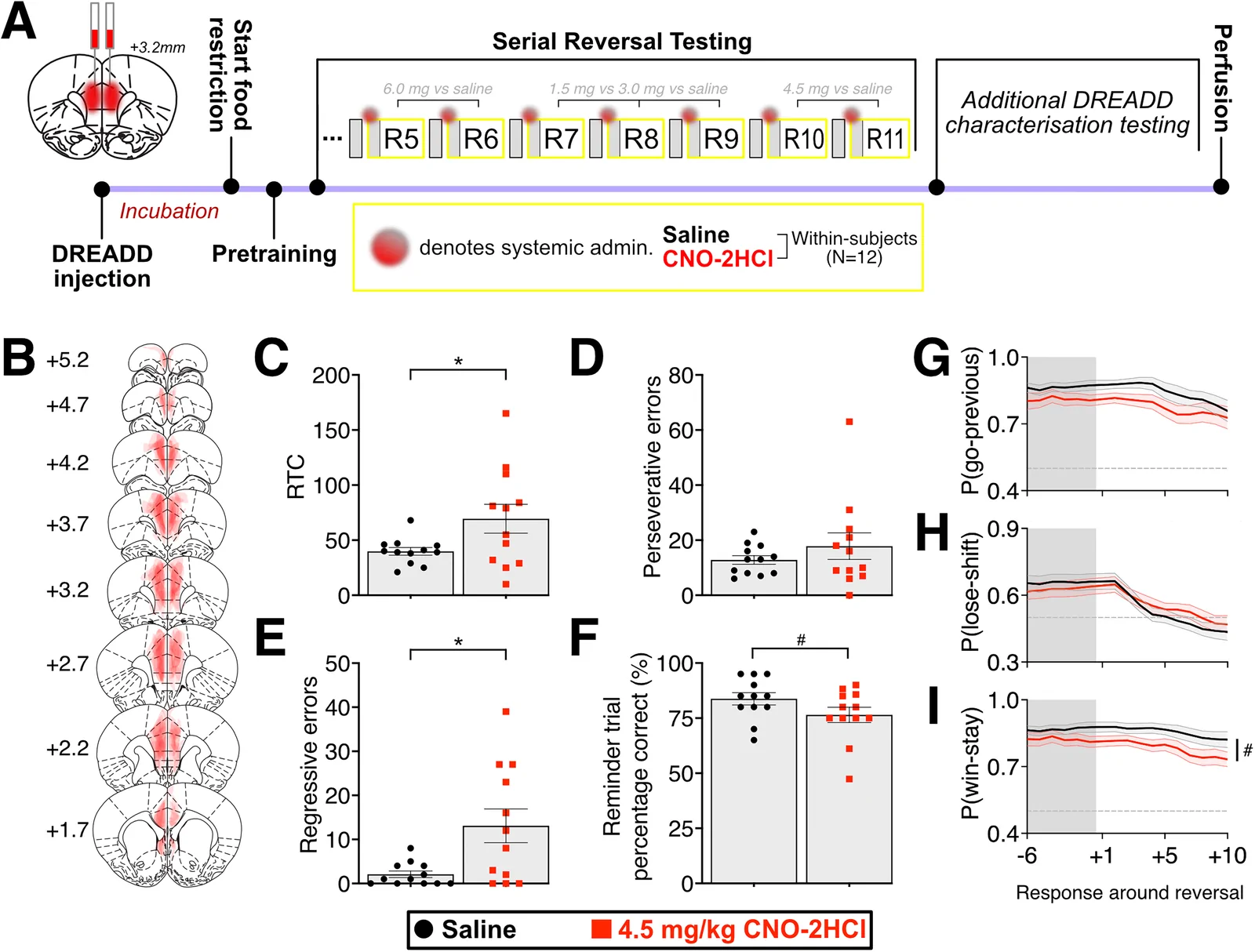
Reversal experiment 3: chemogenetic mPFC disinhibition impaired late reversal learning. ***A***, Timeline of the experiment examining the impact of chemogenetic mPFC disinhibition on late reversal learning. Vgat-Cre rats (*n* = 12) underwent surgery to inject the inhibitory hM4Di DREADD into the mPFC, followed by a 28 d incubation period to allow for expression of the DREADD in GABAergic mPFC neurons, before undergoing food restriction and pretraining on the operant reversal task up to reversal 4. The impact of CNO-2HCl and saline injection was then compared within subjects across reversals 5–11 (R5–11). Several doses of CNO-2HCl were tested, but only the findings following 4.5 mg/kg are shown in this figure. Following reversal testing, rats were used for additional characterization of DREADD functionality in the mPFC (locomotor activity *n* = 12; in vivo electrophysiology *n* = 5; Fig. 3). ***B***, DREADD (mCherry) spread across mPFC. For each individual rat, the area showing mCherry signal was uniformly shaded (red) on coronal sections taken from the brain atlas by Paxinos and Watson (1998). The shadings were overlaid for all rats, meaning darker red areas indicate overlap of DREADD expression across rats. Distance from bregma (in mm), according to the atlas, is indicated on the left. ***C–F***, RTC (***C***), perseverative errors (***D***), regressive errors (***E***), and percentage of correct responses during reminder trials (***F***) following systemic injection of saline (black circles) or CNO-2HCl (4.5 m/kg; red squares). Bar graphs show mean ± SEM, with individual values for each rat plotted to show the range of data. Significant differences and trends as revealed by post hoc pairwise comparisons are indicated as follows: *p < 0.05; ^#^*p* = 0.078. ***G–I***, Probabilities (mean ± SEM) of different strategies are shown for the six reminder-trial responses (gray shading) preceding reversal and the 10 responses following reversal: go-previous (***G***), lose-shift (***H***), and win-stay (***I***). Horizontal dashed line indicates *p* = 0.5 (i.e., chance). The trend toward a main effect of drug for win-stay is indicated by a horizontal line and “^#^” next to data lines (*p* = 0.087).

Reversal experiment 2 showed that mPFC disinhibition by picrotoxin markedly impaired late reversal learning. To confirm and extend this finding, we examined the impact of chemogenetic mPFC disinhibition, by direct inhibition of mPFC GABAergic neurons through the inhibitory DREADD hM4Di, on late reversal learning (Fig. 3*A*). In all rats (*n* = 12; Table 1, Cohort #4), the DREADD was expressed within the mPFC, including the prelimbic, infralimbic, and anterior cingulate cortex, without substantial expression outside the mPFC (Fig. 3*B*). Additional histological, in vivo electrophysiological and behavioral experiments to validate the chemogenetic disinhibition method are outlined in the section following the report of the reversal learning findings.

#### Chemogenetic mPFC disinhibition increased RTC and regressive errors, but not perseverative errors

Chemogenetic disinhibition of the mPFC during late reversal learning, by injection of 4.5 mg/kg CNO-2HCl, increased RTCs compared with saline injection (*F*_(1,11)_ = 6.638, *p* = 0.026, partial *η*^2^ = 0.376; Fig. 3*C*). Interestingly, chemogenetic mPFC disinhibition did not affect perseverative errors (*F* < 1, partial *η*^2^ = 0.072; Fig. 3*D*), contrasting with the increase of perseverative errors by mPFC picrotoxin (Fig. 2*D*), but only increased regressive errors (*F*_(1,11)_ = 8.680, *p* = 0.013, partial *η*^2^ = 0.441; Fig. 3*E*). In addition, chemogenetic mPFC disinhibition tended to impair the expression of the old rule prior to rule change, as reflected by a trend toward a reduced percentage of correct responses during the reminder trials (*F*_(1,11)_ = 3.783, *p* = 0.078, partial *η*^2^ = 0.256; Fig. 3*F*).

Notably, the late reversal impairment caused by chemogenetic mPFC disinhibition presented itself without a substantial impact on response latencies or omission rate (Table S3). Systemic injection of 4.5 mg/kg of CNO-2HCl only tended to increase latencies following reversal (*F*_(1,10)_ = 3.725, *p* = 0.082, partial *η*^2^ = 0.271), irrespective of response type (*F* < 1, partial *η*^2^ = 0.023), but did not affect response latencies during reminder trials (main effect or interaction involving drug, largest *F*_(1,11)_ = 2.292, *p* = 0.158, partial *η*^2^ = 0.172). No other effect or interaction was observed with respect to omissions or response latencies (largest *F*_(1,11)_ = 2.164, *p* = 0.164, partial *η*^2^ = 0.117).

#### Bayesian strategy analysis: chemogenetic mPFC disinhibition induced subtle impairments in task-pertinent strategy implementation around reversal

We also examined the effect of chemogenetic mPFC disinhibition on the probability of task-pertinent strategies around reversal that we had found to be disrupted by mPFC picrotoxin (Fig. 3*G–I*). Chemogenetic mPFC disinhibition numerically reduced adherence to the old rule (go-previous), which was lower following CNO-2HCl (4.5 mg/kg) compared with saline injection across responses around reversal (six responses before, and 10 after reversal), although this effect was not significant (*F*_(1,11)_ = 3.038, *p* = 0.109, partial *η*^2^ = 0.216; drug × response interaction, *F* < 1, partial *η*^2^ = 0.032; Fig. 3*G*). Chemogenetic mPFC disinhibition subtly changed lose-shift behavior: compared with saline, CNO-2HCl reduced lose-shift probability across the six responses before reversal but increased it across the 10 responses following reversal; the latter reflected that lose-shift probabilities following reversal declined less markedly in the CNO-2HCl condition compared with saline (Fig. 3*H*). This was supported by a significant drug × response interaction (*F*_(15,165)_ = 2.691, *p* = 0.001, partial *η*^2^ = 0.197), although simple main effects analysis did not reveal a significant injection effect at any single response (largest *F*_(1,11)_ = 1,465, *p* = 0.254, partial *η*^2^ = 0.117). Finally, chemogenetic mPFC disinhibition tended to reduce win-stay behavior around reversal (Fig. 3*I*). This was supported by a trend toward a main effect of drug (*F*_(1,11)_ = 3.537, *p* = 0.087, partial *η*^2^ = 0.243), in absence of a drug × response interaction (*F*_(15,165)_ = 1.341, *p* = 0.183, partial *η*^2^ = 0.109).

Overall, mPFC disinhibition by chemogenetically inhibiting GABAergic neurons within the mPFC impaired late reversal learning, replicating our finding following mPFC disinhibition by picrotoxin. Our findings suggest that mPFC disinhibition primarily disrupts late reversal learning by increasing regressive errors, as this increase was evident both following chemogenetic mPFC disinhibition with 4.5 mg/kg of CNO-2HCl and following mPFC picrotoxin. Moreover, mPFC picrotoxin reduced win-stay behavior and mPFC chemogenetic disinhibition tended to have the same effect, suggesting that reduction in this behavior is also important for the disruption of reversal learning by mPFC disinhibition. In addition, mPFC picrotoxin also increased perseverative responding, reduced lose-shift behavior, and increased response latencies and omissions. These effects do not seem to be required for the late reversal impairment, because they were not observed following chemogenetic mPFC disinhibition with 4.5 mg/kg CNO-2HCl, although such chemogenetic mPFC disinhibition caused a similarly marked late reversal learning impairment as mPFC picrotoxin.

#### CNO-2HCl did not affect late reversal performance in control rats

Finally, we completed a control experiment to confirm that CNO did not impair late reversal learning in rats not expressing the DREADD (*n* = 16; Table 1, Cohort #5; data not shown). Comparing late reversal performance following injection of 4.5 mg/kg CNO-2HCl or of saline, we observed no significant differences in RTC, reminder-trial performance, regressive errors, response latencies, or omissions (largest *F*_(1,12)_ = 1.695, *p* = 0.217, partial *η*^2^ = 0.097), and only a trend for CNO-2HCl to increase perseverative errors (*F*_(1,15)_ = 3.305, *p* = 0.089, partial *η*^2^ = 0.189). These findings support that CNO-2HCl does not affect late reversal learning independently of DREADD activation.

### Validation of the chemogenetic disinhibition method: histological, in vivo electrophysiological, and behavioral characterization of hM4Di DREADD expression and functionality in mPFC GABA neurons of Vgat-Cre rats

#### Histological verification of spread, penetrance, and specificity of hm4di expression in Vgat-Cre rats

**Figure 4.**
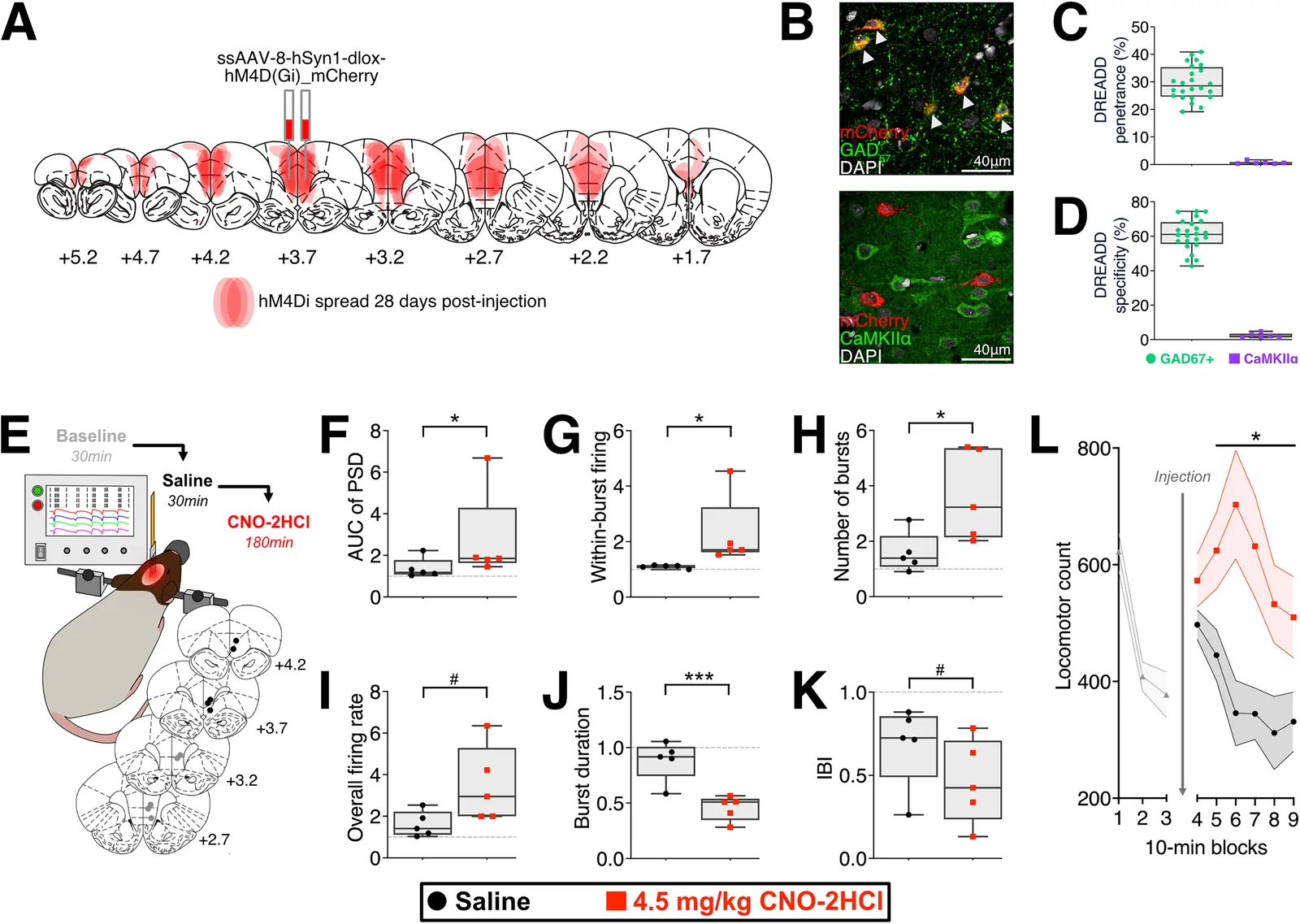
Validation of the chemogenetic disinhibition method: histological, in vivo electrophysiological and behavioral characterization of hM4Di DREADD expression and functionality in mPFC GABA neurons of Vgat-Cre rats. ***A***, DREADD (mCherry) spread across mPFC following viral vector injection at +3.2 mm. For each individual rat, the area showing mCherry signal was uniformly shaded (red) on coronal sections taken from the brain atlas by Paxinos and Watson (1998). The shadings were overlaid for all rats (*n* = 6), meaning darker red areas indicate overlap of DREADD expression across rats. Distance from bregma (in mm), according to the atlas, is indicated underneath each section. ***B***, Image showing staining for GAD67 (top; green), a marker of GABAergic neurons, and CaMKIIa (bottom; green), a marker of excitatory neurons, alongside mCherry signal (red) indicating hM4Di expression. White arrow tips highlight mCherry (hM4Di) expression in GABAergic cells. In both images, nuclear DAPI staining (white) was used to indicate overall cell count. ***C***, Box and whisker plot (whiskers, min–max; hinges, 25th–75th percentile; line, median) of DREADD penetrance in GAD67+ and CaMKIIa+ cells (i.e., proportion of cells of this type that expressed mCherry, the DREADD tag) in slices taken close to the injection site (approximately +3.2 mm from bregma). Individual dots represent values obtained from a single slice. We used four slices per rat for GAD67 (resulting in 24 values for 6 rats; green circles) and one slice for CaMKIIa (resulting in 6 values for 6 rats; red squares), as the latter was predominantly a control staining to ensure no expression in excitatory cells. ***D***, Box and whisker plot of cell-type specificity of DREADD expression (proportion of total mCherry signal attributable to each neuronal subtype). For explanation of the values shown, see legend to ***C***. ***E***, In vivo electrophysiological procedure (top) and electrode locations (bottom). Anaesthetized rats were unilaterally implanted into the mPFC with electrode arrays consisting of four microwires. Recording began with a 30 min baseline period, followed by an intraperitoneal saline injection and another 30 min recording period. Then, CNO-2HCl (4.5 mg/kg, i.p.) was injected followed by a 180 min recording period. Following recording, the most anterior and most posterior electrode tips were electrolytically marked, and rats were transcardially perfused and brains extracted. Approximate locations of electrode tips (most anterior, gray; most posterior, black) on coronal plates adapted from the atlas by Paxinos and Watson (1998). Distance (in mm) anterior to bregma is indicated according to the atlas. ***F–K***, Chemogenetic mPFC disinhibition enhanced mPFC neural burst firing. Box and whisker plots (whiskers, min–max; hinges, 25th–75th percentile; line, median) show measures of LFP (AUC of PSD) and multiunit activity [within-burst firing rate, number of bursts, overall firing rate, burst duration, and interburst interval (IBI), respectively] following systemic saline (black circles) and CNO-2HCl (4.5 mg/kg; red squares), within-subjects. All values are normalized to baseline. Data points for each rat were averaged across the first six 5 min blocks following saline and CNO-2HCl injection, respectively (30 min postinjection). Significant differences and trends revealed by the Wilcoxon signed-rank test (one-tailed) are indicated as follows: *p < 0.05; ***p < 0.001; ^#^0.05 < *p* > 0.1. ***L***, Chemogenetic mPFC disinhibition increased locomotor activity. Locomotor counts (mean ± SEM) are shown for an initial 30 min baseline period (gray triangles) prior to injection (values are averaged across days, as no significant difference in baseline activity was observed *F* < 1) and for the 60 min period following injection of saline (black circles) or CNO-2HCl (4.5 mg/kg; red squares; *n* = 12 rats, within-subjects design). Asterisk and line indicate the 10 min blocks where saline and CNO-2HCl conditions significantly differed (*p* < 0.05).

We used an initial cohort of Vgat-Cre rats (*n* = 6; Table 1, Cohort #3) to characterize DREADD expression in the mPFC. Visualization of the endogenous mCherry tag of DREADD receptors in the brains of these rats revealed bilateral DREADD expression throughout the mPFC, including the prelimbic, infralimbic, and anterior cingulate cortex (Fig. 4*A*). In most rats, expression was restricted to an area corresponding to approximately +4.7 to +2.2 mm anterior to bregma according to the atlas by Paxinos and Watson (1998). There was some DREADD expression outside the mPFC, primarily within the motor cortex (primary and secondary) between +4.2 and +2.7 mm from bregma and in two cases extending to early ventral and dorsal orbitofrontal areas at +5.2 mm from bregma.

Immunohistochemical staining of the brains of the same rats revealed good DREADD penetrance in GABA neurons (GAD67+ cells) and specificity of expression to GAD67+ cells, compared with cells labeled with the excitatory cell marker CaMKIIa+ (Fig. 4*B–D*). As to penetrance, 29.58 ± 1.23% (mean ± SEM) of all GAD_67_+ cells within the slice most proximal to the injection site (i.e., around +3.2 mm from bregma) expressed the DREADD, compared with 0.67 ± 0.19% of all CaMKIIα cells (Fig. 4*C*). As to cell-type specificity, 60.8 ± 1.81% (Fig. 4*D*) of all DREADD expression was localized to GAD_67_+ cells, whereas only 2.35 ± 0.52% was localized in CaMKIIa+ cells. Inspection of CaMKIIa colocalization revealed that proximity of mCherry-expressing GABA+ cells to CaMKIIa+ cells led to the software misclassifying several CaMKIIa+ cells as mCherry+, driving the negligible mCherry-CaMKIIa colocalization. In contrast, GABA and mCherry classification was not affected by this, as overlap for these markers was substantial, reflecting true colocalization. Finally, there was some coexpression of mCherry in ChAT+ cells (data not shown), with an average colocalization of 5.33 ± 0.89%, which, upon closer inspection, was true mCherry expression in ChAT+ cells, probably reflecting a subpopulation of GABA-acetylcholine coreleasing neurons (Saunders et al., 2015; Granger et al., 2016). The remaining difference between total DREADD expression, i.e., the difference between 100% and all cell-type-specific expression (adding up to only ∼70%), likely reflects underestimation of GABA cells, due to either incomplete staining or false-negative classification by the analysis software. Overall, our histological analyses revealed good penetrance and cell-type specificity, consistent with values reported in previous studies using cell-type-specific DREADD expression (Nguyen et al., 2014; Smith et al., 2016).

#### Chemogenetic mPFC disinhibition enhanced mPFC neural burst firing

The neural effects of activating the DREADD receptors expressed within the mPFC were initially examined using 6.0 mg/kg CNO-2HCl (*n* = 5, Table 1, Cohort #3). A notable intensification in burst firing and increased LFP power were observed. However, as indicated in the Materials and Methods, 6.0 mg/kg caused marked nonspecific behavioral effects during reversal testing, and we used a dose of 4.5 mg to study specific effects on late reversal learning. Therefore, in a subset of the rats that had completed the reversal testing (and locomotor testing, see below; *n* = 5; Table 1, Cohort #4), we reexamined the effects of CNO-2HCl on mPFC neural activity at a dose of 4.5 mg/kg. All electrodes were included in the analysis and were placed within the mPFC between +4.7 and +2.7 mm anterior to bregma (Fig. 4*E*), surrounded by strong DREADD expression (Fig. 3*B*). A comparison of baseline mPFC LFP and multiunit measures in isoflurane-anesthetized Vgat-Cre rats in the present study to those that we recorded previously in isoflurane-anesthetized Lister hooded rats (Pezze et al., 2014) indicated overall good agreement (for further details, see Supplemental Results).

Similar to our previous findings following mPFC picrotoxin (Pezze et al., 2014), chemogenetic disinhibition of the mPFC by 4.5 mg/kg of CNO-2HCl increased LFP power, measured as AUC of PSD (*W* = 0, *z* = −2.023, *p* = 0.031, *r*_b_ = −1.000; Fig. 4*F*), and intensified neuronal burst firing, reflected by increased within-burst firing rate (*W* = 0, *z* = −2.023, *p* = 0.031, *r*_b_ = −1.000; Fig. 4*G*) and number of bursts per 5 min block (*t*_(4)_ = 2.807, *p* = 0.024, *d* = −1.255; Fig. 4*I*); overall firing rate also tended to be increased (*W* = 1, *z* = −1.753, *p* = 0.063, *r*_b_ = −0.867; Fig. 4*H*). Additionally, a reduction in burst duration (*t*_(4)_ = 9.848, *p* < 0.001, *d* = 4.404; Fig. 4*J*) and trend for a reduced interburst interval (*t*_(4)_ = 2.017, *p* = 0.057, *d* = 0.902; Fig. 4*K*) was also observed. Overall, our in vivo electrophysiological measurements support that chemogenetic mPFC disinhibition causes neural effects, especially enhanced burst firing, similar to mPFC disinhibition by picrotoxin (Pezze et al., 2014).

#### Chemogenetic mPFC disinhibition increased locomotor activity

In the Vgat-Cre rats used in the main reversal study (*n* = 12; Table 1, Cohort #4), we also examined the locomotor effects of chemogenetic mPFC disinhibition by 4.5 mg/kg CNO-2HCl. Consistent with the locomotor hyperactivity caused by mPFC disinhibition by picrotoxin (Pezze et al., 2014), chemogenetic disinhibition of the mPFC increased open-field locomotor activity (Fig. 4*L*). ANOVA of postinjection locomotor counts revealed a significant drug × 10 min block interaction (*F*_(2.837,31.203)_ = 3.659, *p* = 0.025, partial *η*^2^ = 0.250). Simple main effects analysis of the drug effect at the different 10 min blocks following injection revealed that, compared with saline, CNO-2HCl injection increased locomotor activity across most of the 60 min postinjection (lowest *F*_(1,11)_ = 6.901, *p* = 0.024, partial *η*^2^ = 0.386) period, except for the first 10 min point (*F*_(1,11)_ = 2.711, *p* = 0.128, partial *η*^2^ = 0.198). As expected, locomotor counts during the three 10 min baseline blocks prior to injection did not differ between injection conditions (main effect or interaction involving injection, *F* < 1, partial *η*^2^ = 0.042); there was only a main effect of 10 min block (*F*_(1.353,14.883)_ = 38.435, *p* < 0.001, partial *η*^2^ = 0.777), reflecting locomotor habituation. Finally, there was no carryover effect of mPFC chemogenetic disinhibition on locomotor activity during the 30 min test sessions on the days following the injection days (main effect or interaction involving drug, both *F* < 1, partial *η*^2^ = 0.060), with only the main effect of time persisting (*F*_(2.174,23.917)_ = 56.397, *p* < 0.001, partial *η*^2^ = 0.792; data not shown).

## Discussion

mPFC functional inhibition by muscimol and disinhibition by picrotoxin had distinct effects on early and late reversals. mPFC functional inhibition impaired early reversal performance, tending to increase RTC (Fig. 1*C*) and increasing perseverative errors (Fig. 1*D*) during R2 but did not markedly affect late reversal performance. This early reversal impairment was associated with increased use of go-previous (Fig. 1*G*) and reduced lose-shift (Fig. 1*H*), but intact win-stay (Fig. 1*I*), behavior following reversal. In contrast, mPFC disinhibition reduced RTC at R2, likely due to impaired expression of the previous response and markedly disrupted late reversal performance, increasing RTC (Fig. 2*C*) and both perseverative and regressive errors (Fig. 2*D*,*E*). This late reversal impairment presented alongside the impaired expression of the old response during reminder trials (Fig. 2*F*). It was associated with impaired win-stay and lose-shift behavior (Fig. 2*I*,*H*) and reduced use of the old strategy around reversal (Fig. 2*G*). Next, we used Cre-dependent expression of the inhibitory hM4Di DREADD in the mPFC of Vgat-Cre rats to selectively inhibit mPFC GABAergic neurons. Importantly, chemogenetic mPFC disinhibition also markedly impaired reversal performance, increasing RTC (Fig. 3*C*) and regressive errors (Fig. 3*E*), and tending to reduce win-stay behavior (Fig. 3*I*), without affecting perseverative errors and lose-shift behavior after reversal. We verified that the DREADD was predominantly expressed in GABAergic neurons and showed that DREADD activation particularly enhanced mPFC burst firing and increased locomotor activity (Fig. 4).

### mPFC functional inhibition impaired early, but not late, reversals

mPFC functional inhibition impaired early reversal learning, specifically at R2, but otherwise left reversal performance intact. This is consistent with previous findings that mPFC inactivation did not affect reversal performance at R1 (Floresco et al., 2008) or at reversal stages after R2 (Hervig et al., 2020; these studies did not examine R2). Strikingly similar to our findings, one study reported that cytotoxic mPFC lesions selectively impaired performance at R2 on a two-odor discrimination task, delaying rats’ ability to overcome the early reversal cost at R2, without affecting performance at subsequent reversal stages (Kinoshita et al., 2008). These findings indicate that R2 is qualitatively distinct from preceding and subsequent reversals. R2 is the first time when rats must overcome a previously reinforced reversal response (i.e., return to a response that was initially rewarded, then became unrewarded, and is now rewarded again). This likely requires response control based on an integration of reversal history to infer a higher-order task structure where contingencies alternate repeatedly, rather than simply extinguishing a previously learned response, possibly placing greater demand on mPFC-dependent cognitive control.

mPFC functional inhibition increased perseverative errors and decreased lose-shift behavior at R2. Similarly, reversal impairments caused by mPFC lesions on a three-lever spatial discrimination task (Kosaki and Watanabe, 2012) or by mPFC functional inhibition on a plus-maze reversal task (Avigan et al., 2020) mainly reflected increased perseverative errors. In the present study and on deterministic bowl-digging reversal tasks (Wang et al., 2019), reduced lose-shift behavior was associated with impaired reversal or set-shift performance. On probabilistic variations of the two-lever spatial-reversal task, where only 80% of correct choices were rewarded, mPFC functional inhibition, specifically within the prelimbic cortex, also reduced lose-shift behavior (Dalton et al., 2016; Verharen et al., 2020). However, interestingly, this was not always accompanied by impaired reversal performance, and in Dalton et al. (2016) actually improved reversal performance, possibly reflecting an adaptive advantage to perseveration under conditions of outcome uncertainty. Verharen et al. (2020) noted the different impact of reduced lose-shift behavior on reversal performance across probabilistic reversal experiments, attributing it to baseline differences in win-stay behavior, which correlate with varied task proficiency. Nevertheless, current findings align with previous findings that the mPFC can contribute to early reversal performance by suppressing perseverative errors and sustaining lose-shift behavior.

The mPFC may contribute to reversal learning by other cognitive mechanisms. First, mPFC lesions impaired a touch-screen visual-pattern reversal task mainly by increasing regressive errors and only when difficult to discriminate visual stimuli were used; this was suggested to reflect that the mPFC is important for visual attention (Bussey et al., 1997; Chudasama and Robbins, 2003; Brigman and Rothblat, 2008) and may also reflect the importance of the mPFC for attentional sets (Birrell and Brown, 2000; Brown and Bowman, 2002). However, attentional impairment is unlikely to explain the early reversal learning impairments caused by mPFC functional inhibition in the present study. This is because mPFC functional inhibition selectively increased perseverative errors, whereas an increase in regressive errors would be expected on the basis of an attentional impairment (Bussey et al., 1997; Brigman and Rothblat, 2008); moreover, although both mPFC functional inhibition and disinhibition impaired attention (Pezze et al., 2014; Auger et al., 2017), only functional inhibition impaired early reversal learning.

Second, some studies reported that the mPFC was required for late spatial-reversal learning on plus-maze tasks (but see Rich and Shapiro, 2007; Young and Shapiro, 2009), and this was suggested to reflect that the mPFC is required for working memory (Kidder et al., 2024) and for reducing proactive interference based on hippocampus-dependent spatial memory (Guise and Shapiro, 2017; Avigan et al., 2020). These cognitive mechanisms may also contribute to performance on our two-lever reversal task. However, disruption of these processes cannot easily account for the specific impairments at R2 following mPFC functional inhibition. Finally, the infralimbic mPFC has been implicated in learned irrelevance and inhibitory learning, whereby nonreinforced presentation of a stimulus inhibits subsequent learning about this stimulus (Piantadosi and Floresco, 2014; Lingawi et al., 2018). However, although such processes may be relevant to reversal learning, their disruption would be expected to facilitate learning about the previously nonreinforced lever and reversals and, therefore, cannot account for impaired reversal performance.

### mPFC disinhibition impaired late, but not early, reversals

mPFC disinhibition, by the GABA-A receptor antagonist picrotoxin or chemogenetic inhibition of GABAergic neurons, markedly impaired late reversal learning. Both types of mPFC disinhibition increased RTC and regressive errors and tended to decrease win-stay behavior. In a previous study, pharmacological mPFC disinhibition also reduced win-stay behavior on a reward-discounting task (Piantadosi et al., 2016). This suggests that a key effect of mPFC disinhibition is impaired win-stay behavior. In addition, mPFC picrotoxin impaired lose-shift behavior, increased perseverative errors, and increased response latencies and omissions. These additional effects of mPFC picrotoxin were not critical for the late reversal impairment, which was similarly pronounced following mPFC picrotoxin or chemogenetic disinhibition.

Contrasting with the marked late reversal impairments, mPFC disinhibition by picrotoxin did not disrupt early reversals, consistent with previous work (Enomoto et al., 2011), and even reduced RTC at R2. The latter was at least partly due to a high number of omissions, which limited reminder-trial responses and reduced reinforcement of the old rule, rather than improved behavioral flexibility.

### Distinct effects of mPFC functional inhibition and disinhibition

Electrophysiological recordings showed that mPFC functional inhibition and neural disinhibition had distinct effects on local neural activity: mPFC disinhibition intensified burst firing, whereas functional inhibition reduced firing, including burst firing, of mPFC neurons (Pezze et al., 2014; present study). Despite the distinct neural effects, both mPFC functional inhibition and disinhibition impaired attention (Pezze et al., 2014; Auger et al., 2017). Consequently, we may have expected both manipulations to impair reversal learning. However, the observed double dissociation of the effects of mPFC inhibition and disinhibition on early and late reversal learning highlights that distinct cognitive functions can show distinct relationships to mPFC activity and that mPFC disinhibition can disrupt functions that do not require the disinhibited region, presumably because disinhibition disrupts processing in mPFC projection sites (Bast et al., 2017).

Although the downstream effects of mPFC disinhibition remain to be investigated, several mPFC projection sites may mediate the late reversal impairments by mPFC disinhibition, including the OFC (Sesack et al., 1989), dorsal and ventral striatum (Mailly et al., 2013), and lateral habenula (Baker et al., 2015). The OFC, especially lateral OFC, is required for late reversal learning (Murray et al., 2007; Hervig et al., 2020). The striatum is also required for reversal learning, with dorsomedial striatal lesions impairing late reversals by increasing perseveration (Clarke et al., 2008; Castañé et al., 2010; Young et al., 2023), whereas ventral striatal (nucleus accumbens shell) inactivation impairs late reversals by reducing win-stay behavior (Dalton et al., 2014). Finally, the habenula has been implicated in behavioral flexibility; lateral habenula functional inhibition impaired late T-maze reversals, increasing regressive errors, reducing win-stay behavior, and increasing lose-shift behavior (Baker et al., 2015).

### Conclusion and clinical implications

Our findings suggest that bidirectional mPFC activity changes cause distinct stage-dependent impairments in reversal learning. Reduced mPFC activation can impair early reversal learning by disrupting lose-shift behavior necessary to overcome the early reversal cost, whereas mPFC disinhibition can disrupt late reversals, especially by disrupting win-stay behavior, possibly due to aberrant drive of mPFC projection sites. Considering the functional similarities between rodent mPFC and primate dlPFC (see Introduction), our findings suggest that dlPFC disinhibition and hypofrontality could both contribute to reversal learning deficits, which characterize many neuropsychiatric disorders (Izquierdo et al., 2017). This is particularly relevant to schizophrenia, which has been associated with both dlPFC disinhibition and hypofrontality (see Introduction).
